# A Terphenyllin Derivative CHNQD-00824 from the Marine Compound Library Induced DNA Damage as a Potential Anticancer Agent

**DOI:** 10.3390/md21100512

**Published:** 2023-09-27

**Authors:** Xi-Zhen Cao, Bo-Qi Zhang, Cui-Fang Wang, Jun-Na Yin, Waqas Haider, Gulab Said, Mei-Yan Wei, Ling Lu

**Affiliations:** 1 Key Laboratory of Marine Drugs, The Ministry of Education of China, School of Medicine and Pharmacy, Ocean University of China, Qingdao 266003, China; caoxizhen2022@163.com (X.-Z.C.); zbq2867653686@163.com (B.-Q.Z.); wangcuifang0115@163.com (C.-F.W.); yinjunna@163.com (J.-N.Y.); waqashaider07@yahoo.com (W.H.); gulabouc@gmail.com (G.S.); mywei95@126.com (M.-Y.W.); 2 Laboratory for Marine Drugs and Biological Products, Laoshan Laboratory, Qingdao 266003, China; 3 Department of Chemistry, Women University Swabi, Swabi 23430, Pakistan

**Keywords:** terphenyllin derivatives, marine compound library, cell cycle, apoptosis, anti-cancer

## Abstract

With the emergence of drug resistance and the consequential high morbidity and mortality rates, there is an urgent need to screen and identify new agents for the effective treatment of cancer. Terphenyls—a group of aromatic hydrocarbons consisting of a linear 1,4-diaryl-substituted benzene core—has exhibited a wide range of biological activities. In this study, we discovered a terphenyllin derivative—CHNQD-00824—derived from the marine compound library as a potential anticancer agent. The cytotoxic activities of the CHNQD-00824 compound were evaluated against 13 different cell lines with IC_50_ values from 0.16 to 7.64 μM. Further study showed that CHNQD-00824 inhibited the proliferation and migration of cancer cells, possibly by inducing DNA damage. Acridine orange staining demonstrated that CHNQD-00824 promoted apoptosis in zebrafish embryos. Notably, the anti-cancer effectiveness was verified in a doxycin hydrochloride (DOX)-induced liver-specific enlargement model in zebrafish. With Solafinib as a positive control, CHNQD-00824 markedly suppressed tumor growth at concentrations of 2.5 and 5 μM, further highlighting its potential as an effective anticancer agent.

## 1. Introduction

**Figure 1:**
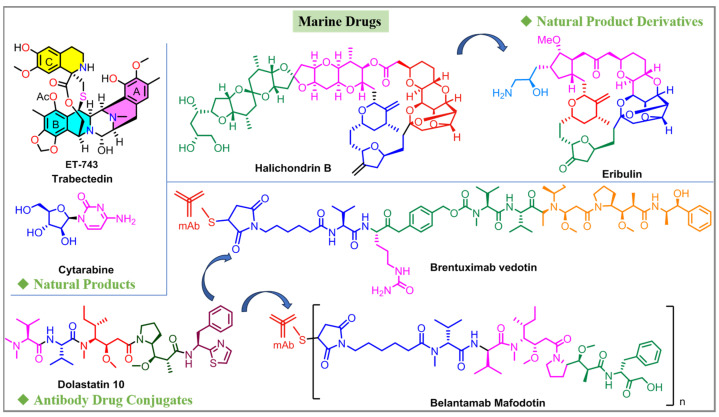
Marine natural products approved as the anticancer drugs.

Cancer is a prominent global public health issue and remains one of the primary causes of death worldwide. It is predicted that the incidence of all cancers combined will double by 2070 relative to 2020 [1]. In recent years, the COVID-19 pandemic has placed a significant burden on medical resources, impacting the diagnosis and treatment of cancer. This has led to an increase in advanced cancer cases and mortality rates [2,3]. Due to the emergence of drug resistance and high morbidity and mortality, there is an urgent need to discover new anticancer drugs. Natural products are important resources for drug development. Over the past 40 years, approximately 1400 small molecule drugs have been approved, with over two-thirds of them being related to natural products [4]. Therefore, natural products and their derivatives play important roles in drug development. The unique environment of the ocean has created complex, novel, and diverse marine natural products, making them an important component of natural products [5,6]. Marine secondary metabolites with novel structures and diverse biological activities have been demonstrated to be rich sources of chemical entities for drug discovery [7,8,9,10,11,12]. At present, among the more than 36,000 reported marine natural products, 17 have been approved for therapeutic use worldwide, especially in the field of anticancer drug research [13]. Figure 1 shows the marine natural products that have been approved as anticancer drugs. Trabectedin, derived from *Ecteinascidia turbinate*, is a marine natural product that is clinically used for the treatment of advanced soft tissue sarcoma [14]. Eribulin, on the other hand, originated from the marine natural product halichondrin B [15]. Cytarabine is another marine drug developed from a marine natural product [16]. In addition, the current widely used strategies and emerging technologies, such as natural product-based antibody drug conjugates (ADCs) like brentuximab vedotin and brentuximab mafodotin, also originated from the marine natural product dolastatin 10 [17,18]. Therefore, marine natural products have consistently proved to be valuable resources for developing anticancer leads.

Investigating new bioactive natural products and their derivatives from marine fungi is a significant and ongoing research focus in our laboratory [19,20,21,22,23]. As part of our continuous efforts to search for biologically active secondary metabolites from marine-derived fungi, terphenyllin was obtained from *Aspergillus* sp. [24]. Terphenyls—a group of aromatic hydrocarbons consisting of a linear 1,4-diaryl-substituted benzene core—exhibited a wide range of biological activities, including cytotoxicity, α-glucosidase inhibitory activity, and AMPK activator activity [23,24,25,26]. Interestingly, some of them showed strong cytotoxic activity against hepatocellular liver carcinoma cell lines (HepG2 and SMMC-7721), which were equivalent or stronger than the positive controls, Adriamycin and fluorouracil [24,27]. To date, more than 230 *p*-terphenyls have been isolated from microorganisms such as lichen endophytes, actinomycetes, and mosses [28]. The earliest chemical studies of *p*-terphenyls as one class of the pigments of mushrooms were carried out in 1877 [28]. In particular, one *p*-terphenyl derivative—MK-8722, designed by Merck—was developed as a hypoglycemic drug lead [29]. This further confirmed the significance and utility of *p*-terphenyl derivatives. Based on previous research, approximately 120 terphenyllin derivatives were designed and synthesized, which created a marine terphenyl derivatives library. Among them, two compounds—Terphenyllin and 3′-(isopentyloxy)-2′,5′-dimethoxy-[1′,1′:4′,1′-terphenyl]-4,4″-diol—not only showed strong *α*-glucosidase inhibitory activity, but also had relatively higher therapeutic indices, with the potential to be promising leads [24]. Additionally, eight compounds were also found to have cytotoxic potency [24,25]. Therefore, these compounds have shown value for further in-depth research.

**Figure 2:**
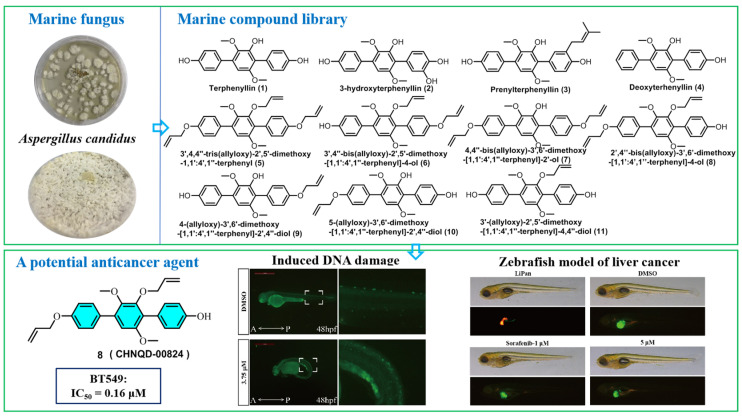
The discovery and biological activity research of compound CHNQD-00824.

In the present study, we scanned and identified a terphenyllin derivative—CHNQD-00824—derived from the marine-derived compound library as a potential anticancer agent. Furthermore, thirteen different cancer cells were selected for activity screening. Further study showed that this compound can inhibit the proliferation and migration of cancer cells by inducing DNA damage. CHNQD-00824 could inhibit DOX-induced liver-specific enlargement. The present results provided evidence that CHNQD-00824 exhibited great potential to be developed as an anticancer agent (Figure 2).

## 2. Results and Discussion

### 2.1. Chemistry

**Figure 3:**
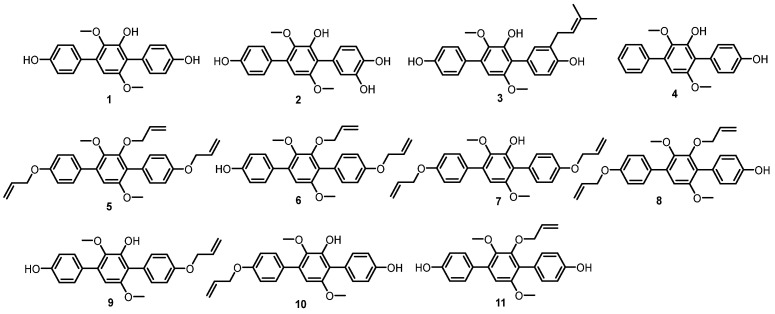
Natural product compounds **1**–**4** and terphenyllin derivatives **5**–**11**.

**Scheme 1:**
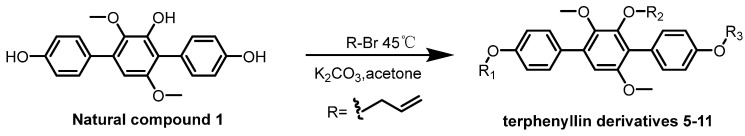
Strategy for the semi-synthesis of terphenyllin derivatives **5**–**11**.

Four terphenylated natural products, **1**–**4**, and seven terphenyllin derivatives, **5**–**11**, were scanned for their activity against BT549 cell lines from the marine-derived compound library. The four terphenylated natural products were identified from the marine fungus *Aspergillus candidus* (CHNSCLM-0393). Compound **1**, which contained three phenolic hydroxyl groups, was identified as terphenyllin [30]. Compounds **2**–**4** were identified as 3-hydroxyterphenyllin, prenylterphenyllin, and deoxyterhenyllin, respectively [31,32,33,34]. Seven terphenyllin derivatives, **5**–**11**, were obtained through the etherification reaction (Scheme 1). The etherification reaction was carried out at 45 °C for 2~3 h with dry K_2_CO_3_ as a catalyst and acetone as a solvent. Compound **5** was the terphenyllin trisubstituted derivative with all three phenolic hydroxyl groups alkylated, whereas compound **6** was the di-substituted derivative with allyl substituents at the R_2_ and R_3_ positions. Their structures were determined through nuclear magnetic resonance (NMR) analysis and ESIMS (Figures S1–S33). Their structures are shown in Figure 3.

In view of the potential biological activity of terphenyllin derivatives, we investigated the fermentation conditions of this fungus, including the effects of solid and liquid media, as well as the salinity, to increase the production of compound **1**. The liquid medium was PDB medium, which consisted of 200 g potato, 50 g glucose plus water to 1 L, adding sea salt according to the salinity, and the solid medium was rice with 4% artificial seawater. We set the salinity of the fermentation conditions as 1%, 2%, 4%, and 8%. The results showed that the yield of compound **1** was much better under solid fermentation conditions than under liquid fermentation conditions and that solid fermentation conditions are much better than liquid fermentation conditions. Among the currently set culture media, the solid culture medium with a salinity of 4% was the condition that obtained the highest yield of compound **1** (Figure S34). Accordingly, the fermentation conditions were solid rice media in 1 L Erlenmeyer flasks. Each flask contained 65 g of rice and 70 mL of PDB medium at 4% salinity for 35 days at room temperature.

### 2.2. Biological Evaluation

#### 2.2.1. Cytotoxic Activity

**Figure 4:**
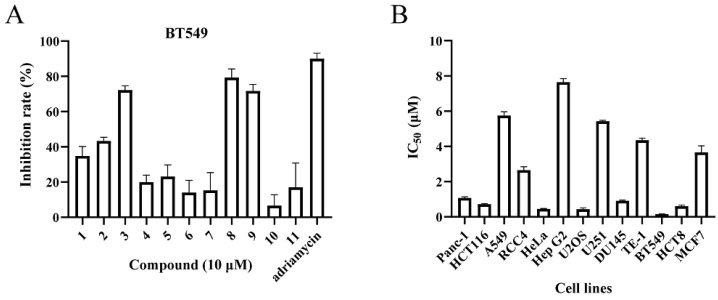
The cytotoxicity data of compounds **1**–**11**. (**A**) The inhibitory activity of compounds **1**–**11** against BT549 cell line. Adriamycin as a positive control. (**B**) CHNQD-00824 evaluated in thirteen different tumor cells. *n* = 3 biological replicate samples.

In this study, we first screened cytotoxic active compounds from the marine compound library using 2-(2-methoxy-4-nitrophenyl)-3-(4-nitrophenyl)-5-(2,4-disulfobenzene)-2H-tetrazolium monosodium salt CCK8 assays. Four terphenylated natural products, **1**–**4**, and seven terphenyllin derivatives, **5**–**11**, were further scanned for their activity against BT549 cell lines from the marine-derived compound library (Figure 4A). Terphenyllin derivative **8** (CHNQD-00824) was discovered as a potential anticancer agent. Furthermore, this compound was evaluated against a panel of thirteen different cancer cells, including human pancreatic cancer (Panc-1), colon cancer (HCT116), non-small cell lung cancer (A549), renal cancer (RCC4), cervical cancer (HeLa), liver cancer (HepG2), osteosarcoma (U2OS), glioma (U251), prostate cancer (DU145), esophageal cancer (TE-1), breast cancer (BT549, MCF7), and caecal adenocarcinoma (HCT8) cell lines using the CCK8 assay. As shown in Figure 4, CHNQD-00824 exhibited broad-spectrum cytotoxic activities against various cell lines, with IC_50_ values ranging between 0.16 and 7.64 μM (Table 1). Notably, it showed potent inhibitory activity against BT549, U2OS, HCT8, HCT116, and DU145 cells, with IC_50_ values in the sub-micromolar range. These findings suggest that CHNQD-00824 has significant potential for development as an anticancer agent. The above studies showed that, compared with the natural product terphenyllin **1**, only two derivatives—CHNQD-00824 and **9**—exhibited stronger cytotoxic activity. The introduction of allyl groups through alkylation reactions could affect the cytotoxic activity. In particular, when both the R_1_ and R_2_ positions were replaced by allyl groups, or when the allyl group was introduced only at the R_3_ position, the cytotoxic activity was significantly enhanced.

#### 2.2.2. CHNQD-00824 Inhibited the Proliferation and Migration of Breast Cancer Cells

**Figure 5:**
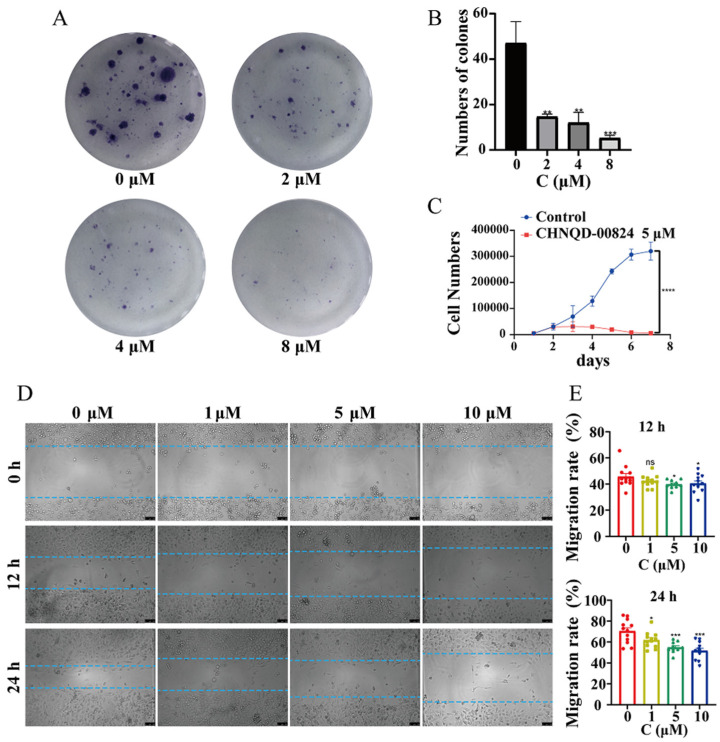
CHNQD-00824 inhibited the proliferation and migration of BT549 cells. (**A**,**B**) CHNQD-00824 inhibited the colony formation of BT549 cells. The BT549 cells were treated with CHNQD-00824 for 13 d, then the clones were photographed and counted. (**C**) Growth curves of BT549 cells treated with CHNQD-00824 (5 μM) and DMSO. (**D**,**E**) The scratch assay of BT549 cells after treatment with CHNQD-00824 for 12 h and 24 h. Scale bar, 100 μm. The data shown are the mean ± SD (* *p* < 0.05, ** *p* < 0.01, *** *p* < 0.001, **** *p* < 0.0001 and ns, nonsignificant). *n* = 3 biological replicate samples.

This CHNQD-00824 compound was found to have strong inhibitory activity on BT549 cells, with an IC_50_ value of 0.16 μM. To further investigate its effect on the cell viability and proliferative capacity, single-cell clone formation and cell counting assays were performed [35]. Treatment of BT549 cells with CHNQD-00824 for 13 days dose-dependently reduced the formation of colonies, indicating its anticancer activity. Meanwhile, the cell counting assay showed that CHNQD-00824 at a concentration of 5 μM almost completely inhibited cell proliferation. Increased invasion and cell motility play a critical role in cancer progression [36,37]. Taking this fact into consideration, the anti-migratory activity of CHNQD-00824 against BT549 cells was evaluated using the scratch wound healing assay [38]. The results indicate that after 12 h treatment, there was no significant effect on the cell viability. However, a significant inhibition of cell migration was observed at concentrations of 5 μM and 10 μM. Additionally, CHNQD-00824 exhibited a notable inhibitory effect on cell migration after 24 h of treatment at concentrations of 1, 5, and 10 μM (Figure 5). Collectively, these results indicate that CHNQD-00824 inhibited the proliferation and migration of breast cancer cells in vitro.

#### 2.2.3. CHNQD-00824 Induced G2 Phase Cell Cycle Arrest in BT549 Cells

**Figure 6:**
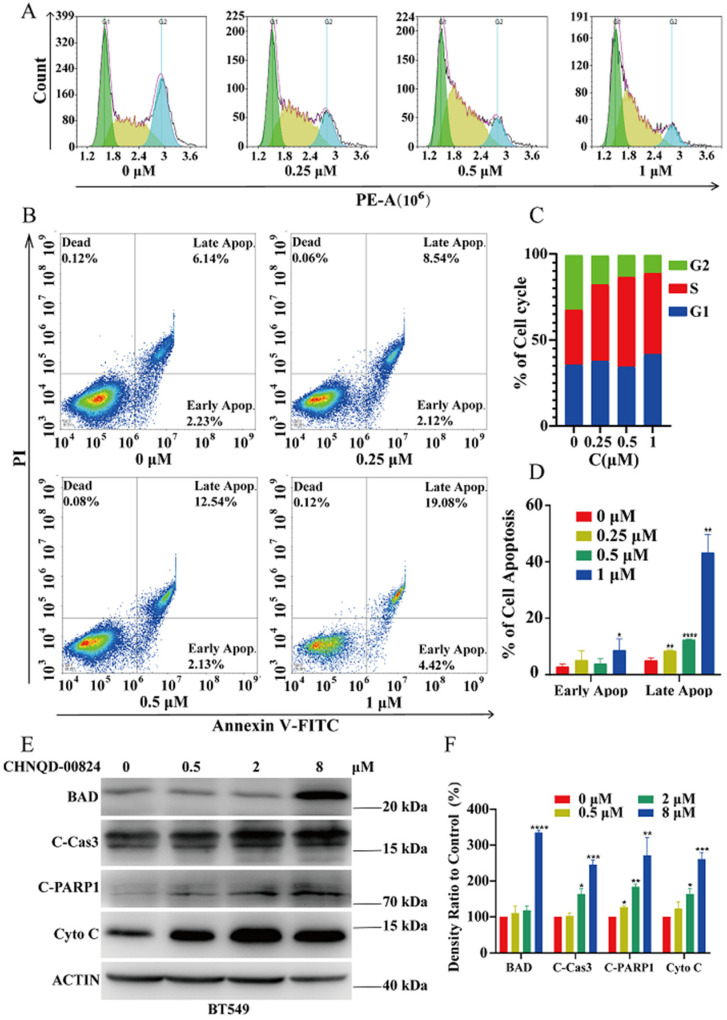
CHNQD-00824-induced apoptosis and cell cycle arrest in BT549 cells. (**A**,**C**) Flow cytometry analysis of cell cycle analysis on BT549 cells after treatment with CHNQD-00824 for 24 h. (**B**,**D**) Flow cytometry analysis of BT549 cells stained with the Annexin V/FITC-PI double staining solution after treatment with CHNQD-00824 for 24 h. (**E**) Effect of CHNQD-00824 on apoptosis-related proteins. BT549 cells were treated with CHNQD-00824 (0–8 μM) for 24 h, then cells were harvested and proteins were separated by electrophoresis on SDS-PAGE. Western blotting analysis were performed to detect the expression of cleaved-PARP, cleaved-caspase-3, Cyto-c and BAD. (**F**) Histogram shows the relative abundance of apoptosis-related proteins compared with the control group. The data shown are the mean ± SD (* *p* < 0.05, ** *p* < 0.01, *** *p* < 0.001, **** *p* < 0.0001, and ns, nonsignificant). *n* = 3 biological replicate samples.

Dysregulation of the cell cycle can lead to uncontrolled cell proliferation and cancer development. In order to further explore the mechanism of CHNQD-00824 inhibiting BT549 proliferation and migration, the cell content was analyzed to identify its impact on the BT549 cell cycle distribution. As shown in Figure 6A,C, the percentage of cells in the G2 phase decreased significantly, from 31.78% to 10.44%, in a dose-dependent manner after treatment with CHNQD-00824. The G2 phase is an important checkpoint in the cell cycle, where DNA damage is repaired before cells proceed to mitosis. Inhibiting the transition of cells from the G2 phase to mitosis can effectively halt cell division and proliferation. The observed G2 phase cell cycle arrest induced by CHNQD-00824 suggests that the compound may interfere with the normal cell cycle progression in BT549 cells. Further studies are needed to elucidate the exact molecular mechanism by which CHNQD-00824 induces G2 phase cell cycle arrest.

#### 2.2.4. CHNQD-00824 Induced Caspase-Dependent Apoptosis in BT549 Cells

Then, we investigated whether CHNQD-00824 could induce the apoptosis of BT549 cells. According to Figure 6B, the percentage of apoptosis cells in the control group was 8.37%. However, the treatment with increasing concentrations of CHNQD-00824 (0.25, 0.5, and 1 μM) resulted in a dose-dependent increase in the percentage of apoptotic cells, reaching 10.66%, 14.67%, and 23.50%, respectively. The percentage of the cell apoptosis distribution is shown in Figure 5D. The result revealed that CHNQD-00824 could significantly induce apoptosis in a concentration-dependent manner. To gain insights into the underlying molecular mechanisms of the apoptosis induction by CHNQD-00824, the expression levels of apoptosis-related proteins were examined using western blotting analysis [39,40,41,42]. Compared to the control group, the treatment with CHNQD-00824 (0.5, 2, 8 μM) led to an increase in the levels of cleaved-PARP1 (C-PARP1) and Cytochrome C (Cyto C). Additionally, CHNQD-00824 was found to increase the level of cleaved-caspase 3 (C-Cas3) at the concentrations of 2 and 8 μM,. Furthermore, the pre-apoptotic protein, BAD, was observed to increase upon treatment with 8 μM of CHNQD-00824. Taken together, the findings indicated that CHNQD-00824 facilitated apoptosis in the BT549 cells (Figure 6E,F).

#### 2.2.5. CHNQD-00824 Caused DNA Damage in Both BT549 Cells and Zebrafish

**Figure 7:**
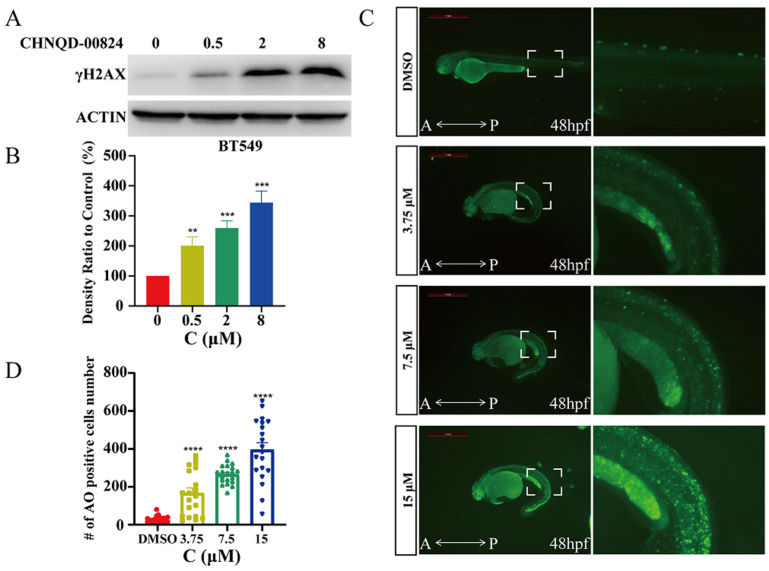
CHNQD-00824-induced DNA damage. (**A**) Effect of CHNQD-00824 on DNA damage marker proteins. BT549 cells were treated with CHNQD-00824 (0–8 μM) for 24 h, then cells were harvested and proteins were separated by electrophoresis on SDS-PAGE. Western blotting analysis were performed to detect the expression of γH2AX. (**B**) Histogram shows the relative abundance of γH2AX compared with the control group. (**C**,**D**) AO staining of zebrafish embryos at 48 hpf. When development reached 24 hpf, zebrafish embryos were treated with CHNQD-00824 (0–15 μM) to 48 hpf. AO staining was then performed. Scale bar, 1 mm. The data shown are the mean ± SD (** *p* < 0.01, *** *p* < 0.001, **** *p* < 0.0001). *n* = 3 biological replicate samples.

Considering that the stability of DNA is closely related to cell apoptosis and the cell cycle, the change in the protein level of phospho-histone H2AX-S139, a marker of DNA damage, was detected [43]. After treatment with CHNQD-00824, the protein level of phospho-histone H2AX-S139 was significantly upregulated (Figure 7A,B). The accumulation of DNA damage might lead to cell death via apoptosis; therefore, we assessed the level of apoptosis using acridine orange (AO) staining in zebrafish in vivo. Fluorescent AO dye staining is an effective method for investigating cell death in zebrafish embryos [44]. The zebrafish embryos at 24 h post-fertilization (hpf) were exposed to CHNQD-00824. Although no significant mortality was observed, developmental toxicity did occur, primarily characterized by a bent spine. The analysis of the AO-stained embryos clearly demonstrated upregulated apoptosis after the CHNQD-00824 treatment (Figure 7C,D). These results implied that CHNQD-00824 induced cell death via DNA damage.

#### 2.2.6. CHNQD-00824 Inhibited DOX-Induced Liver-Specific Enlargement

Traditionally, the murine model has been used in research as an in vivo model organism. Zebrafish, owing to their small size and rapid maturation time, have emerged as an important new cancer model that complements what can traditionally be achieved in mice and cell culture systems. Furthermore, the genetic pathways driving cancer are highly conserved between zebrafish and humans, and the ability to easily manipulate the zebrafish genome to rapidly generate transgenic animals makes zebrafish an excellent model organism [45,46,47]. In recent years, several inducible liver tumor models have been generated through the transgenic expression of oncogene in hepatocytes in zebrafish. In this report, the transgenic line—*Tg* (*fabp10:rtTA2s-M2*; *TRE2:EGFP kras^G12V^*), named *To*(*kras^G12V^*)—was employed to investigate the effect of CHNQD-00824 on hepatocellular carcinoma (HCC). Under the induction of doxycin hydrochloride (DOX), *To*(*kras^G12V^*) exhibited the hepatocyte-specific expression of oncogenic krasV12, and eventually led to abnormal liver enlargement and even liver cancer in zebrafish [2,3,48]. In addition, a two-color transgenic zebrafish line—*Tg* (fabp10:DsRed; elaA:GFP)—with DsRed-labeled liver and GFP-labeled pancreas, was used as a normal control for the liver morphology, and was referred to as LiPan.

**Figure 8:**
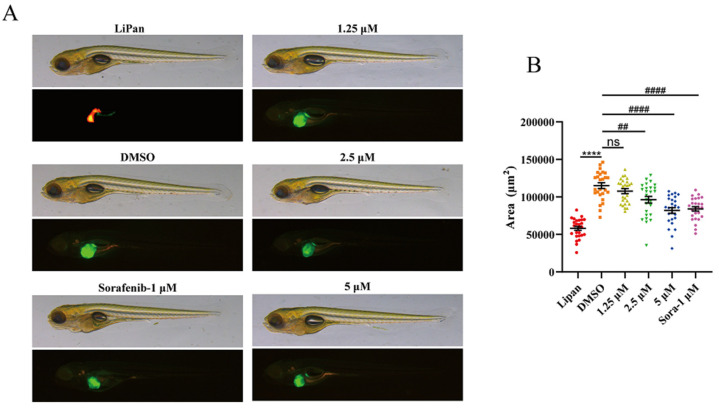
CHNQD-00824 inhibited tumor growth in zebrafish. (**A**) Zebrafish model of liver cancer. When development reached 3 dpf, zebrafish were treated with DOX (60 mg/L) and CHNQD-00824 (1.25–5 μM) to 7 dpf. Sorafenib as positive control. (**B**) Tumor size in zebrafish was measured by photography at 7 dpf. The data shown are the mean ± SD (**** *p* < 0.0001, ^##^ *p* < 0.01, ^####^ *p* < 0.0001, and ns, nonsignificant). *n* = 3 biological replicate samples.

Therefore, the *To*(*kras^G12V^*) transgenic zebrafish model was used to test the effect of CHNQD-00824 on the growth of HCC in vivo. When developed to 3 dpf, the zebrafish were treated with different doses of CHNQD-00824, and DOX was added to induce abnormal liver enlargement. Following the exposure to CHNQD-00824 at this stage, no significant abnormalities or deformities were observed in the treated zebrafish. At 7 dpf, the fluorescence area of the zebrafish liver was observed and analyzed. LiPan zebrafish treated with the same concentration of DMSO were used as a control. The results showed that CHNQD-00824 could dose-dependently inhibit DOX-induced liver-specific enlargement at the concentrations of 1.25 μM, 2.5 μM, and 5 μM (Figure 8), suggesting that CHNQD-00824 has potential as a therapeutic agent for liver cancer.

## 3. Materials and Methods

### 3.1. General Experimental Procedures

NMR spectra were recorded on a Bruker Advance NEO 400; chemical shifts δ are reported in ppm, using TMS as internal standard, and coupling constants (*J*) are in Hz. The HRESIMS and ESIMS spectra were obtained using a Micromass Q-TOF mass spectrometer. UPLC-MS was performed on a Waters UPLC^®^ system (Waters Ltd., Milford, MA, USA) using a C18 column [(Waters Ltd.) ACQUITY UPLC^®^ BEH C18, 2.1 × 50 mm, 1.7 μm; 0.5 mL/min] and ACQUITY QDa ESIMS scan from 150 to 1000 Da. Column chromatography (CC) was performed on silica gel (Qingdao Haiyang Chemical Group Co., Qingdao, China; 200 to 300 mesh) and Sephadex LH-20 (Amersham Biosciences, Amersham, UK). TLC silica gel plates (Yan Tai Zi Fu Chemical Group Co., Yantai, China; G60, F-254) were used for thin layer chromatography. Semi-preparative HPLC was performed on a Waters 1525 system using a semi-preparative C18 column (Amsterdam, Netherlandish; Kromasil, 5 μm, 10 × 250 mm) equipped with a Waters 2996 photodiode array detector, and the flow rate was 2.0 mL/min.

### 3.2. Fungal Material

The fungal strain *A. candidus* (CHNSCLM-0393) was isolated from a piece of fresh internal tissue of the gorgonian coral *Juncella fragilis* collected from the Spratly Islands. The strain was identified through DNA amplification and sequencing of the ITS region according to the molecular biology methods described in the literature [49]. The fungus was identified as *A. candidus* with the GeneBank (NCBI) accession number MF681708.

### 3.3. Fermentation, Extraction, and Isolation

The fungal strain *A. candida* (CHNSCLM-0393) was grown in 60 1 L Erlenmeyer flasks in solid rice medium. Each flask contained 65 g of rice and 70 mL of 4% artificial seawater. The fungal was fermented for 35 days at room temperature. Each flask was extracted three times with 400 mL EtOAc from the fermented solid medium and then concentrated to dryness under vacuum to give an EtOAc extract (50 g). This was separated through column chromatography (CC) on silica gel, Sephadex LH-20 CC, and then recrystallized to give the two main compounds: **1** (5.6 g) and **2** (1.5 g). Further separation through semi-preparative HPLC gave compound **3** (5.0 mg) and compound **4** (8.5 mg). The structures of **1**–**4** were determined by analyzing the NMR data and comparing it with the literature data. The NMR data for natural products **1**–**4** are presented in the supplementary data.

### 3.4. General Synthetic Methods for Compounds ***5**–**11***

The etherification of natural product **1** was performed by alkylating the three hydroxyl groups by introducing allyl bromide to give seven derivatives, **5**–**11**. To an acetone solution (10 mL) of **1** (150 mg, 0.44 mM), 2.5 equivalents of allyl bromide (134.1 mg, 1.11 mM) and 3 dry equivalents of K_2_CO_3_ (183.8 mg, 1.33 mM) were added. The reaction was stirred at 45 °C for 3 h and monitored through TLC. The reaction solution was extracted with EtOAc and the solvent was removed from the organic layer under reduced pressure. The extracts were then separated on silica gel CC (200–300 mesh) using a gradient of petroleum ether-EtOAc from 9:1 to 4:1 (*v*/*v*). Compound **5** (16.2 mg) was obtained through the elution of 500 mL petroleum ether-EtOAc eluent at a 9:1 (*v*/*v*) eluent ratio, and compound **7** (12.8 mg) was obtained through subsequent elution. When the ratio of the petroleum ether-EtOAc eluent was 85:15 (*v*/*v*), compounds **8** and **6** could be obtained, of which compound **6** had a low content, and compounds **8** (18.9 mg) and **6** (4.5 mg) were then obtained using the semipreparative HPLC (80% MeOH-H_2_O). Subsequently, semipreparative HPLC (70% MeOH-H_2_O) was used to obtain compounds **10** (23.5 mg), **9** (5.2 mg), and **11** (7.9 mg), wherein compound **10** was the main reaction product. The unreacted starting compound **1** was finally eluted with a petroleum ether-EtOAc eluent ratio of 4:1 (*v*/*v*).

Their structures were all identified using the NMR data and HRESIMS spectroscopy. Compounds **5** and **6** were new terphenyllin derivatives, and named 3′,4,4″-tris(allyloxy)-2′,5′-dimethoxy-1,1′:4′,1″-terphenyl (**5**) and 3′,4″-bis(allyloxy)-2′,5′-dimethoxy-[1,1′:4′,1″-terphenyl]-4-ol (**6**), respectively. Compounds **7**–**11** were identified using the NMR and ESIMS data, the details of which are added in the supplementary data.

3′,4,4″-tris(allyloxy)-2′,5′-dimethoxy-1,1′:4′,1″-terphenyl (**5**), White, amorphous powder; ^1^H NMR (400 MHz, acetone-*d*_6_) *δ* 7.57 (2H, d, *J* = 8.8 Hz), 7.30 (2H, d, *J* = 8.6 Hz), 7.03 (2H, d, *J* = 8.8 Hz), 6.98 (2H, d, *J* = 8.6 Hz), 6.77 (1H, s), 6.12 (2H, m), 5.78 (1H, m), 5.46 (1H, dd, *J* = 17.2 Hz, 1.8 Hz), 5.27 (1H, dd, *J* = 10.6, 1.8 Hz), 5.13 (1H, dd, *J* = 17.2, 1.8 Hz), 5.01 (1H, dd, *J* = 10.6, 1.8 Hz), 4.64 (2H, d, *J* = 5.2 Hz), 4.62 (2H, d, *J* = 5.2 Hz), 4.28 (2H, d, *J* = 5.2 Hz), 3.74 (3H, s), 3.56 (3H, s); ^13^C NMR (100 MHz, acetone-*d*_6_) *δ* 159.0 (C), 158.6 (C), 154.2 (C), 151.7 (C), 145.8 (C), 135.5 (C), 135.1 (CH), 134.9 (CH), 134.8 (CH), 132.8 (CH × 2), 131.7(C), 131.1 (CH × 2), 127.3(C), 125.2(C), 117.4 (CH_2_), 117.3 (CH_2_), 116.8 (CH_2_), 115.2 (CH × 2), 114.6 (CH × 2), 108.8 (CH), 74.5 (CH_2_), 69.3 (CH_2_), 69.2 (CH_2_), 60.9 (CH_3_), 56.3 (CH_3_). (+)-HR-ESI-MS *m*/*z* 459.2149 [M + H]^+^, (calcd for C_29_H_31_O_5_, 459.2166).

3′,4″-bis(allyloxy)-2′,5′-dimethoxy-[1,1′:4′,1″-terphenyl]-4-ol (**6**); White, amorphous powder; ^1^H NMR (400 MHz, acetone-*d*_6_) *δ* 8.49 (1H, s), 7.48 (2H, d, *J* = 8.8 Hz), 7.30 (2H, d, *J* = 8.8 Hz), 6.98 (2H, d, *J* = 8.8 Hz), 6.93 (2H, d, J = 8.8 Hz), 6.75 (1H, s), 6.12 (1H, ddt, *J* = 17.2, 10.5, 5.2 Hz), 5.78 (1H, ddd, *J* = 22.7, 10.7, 5.5 Hz), 5.45 (1H, dd, *J* = 17.2, 1.8 Hz), 5.27 (1H, dd, *J* = 10.6, 1.5 Hz), 5.13 (1H, dd, *J* = 17.2, 1.8 Hz), 5.01 (1H, dd, *J* = 10.6, 1.5 Hz), 4.62 (2H, dt, *J* = 5.1, 1.4 Hz), 4.28 (2H, dt, *J* = 5.4, 1.3 Hz), 3.73 (3H, s), 3.56 (3H, s); ^13^C NMR (100 MHz, acetone-*d*_6_) *δ* 158.5 (C), 157.8 (C), 154.1 (C), 151.7 (C), 145.8 (C), 135.6 (C), 135.4 (CH), 134.9 (CH), 132.9 (CH × 2), 131.2 (CH × 2), 130.5 (C), 127.4 (C), 125.0 (C), 117.3(CH_2_), 116.8(CH_2_), 115.9 (CH × 2), 114.6 (CH × 2), 108.8 (CH), 74.5 (CH_2_), 69.3 (CH_2_), 60.9 (CH_3_), 56.3 (CH_3_). (+)-HR-ESI-MS *m*/*z* 419.1841 [M + H]^+^, (calcd for C_26_H_27_O_5_, 419.1853).

### 3.5. Biology

#### 3.5.1. Animals

Zerafish (Danio rerio) were maintained on a 14/10 h light/dark cycle at 28 °C and fed twice daily. *To*(*kras^G12V^*) and LiPan mutants were gifts from the laboratory of Zhi-yuan Gong (National University of Singapore). Zebrafish embryos were obtained through natural hybridization. To prevent melanin from affecting the liver cancer, the embryo medium was supplemented with 0.003% (*w*/*v*) 2-phenylthiourea. All experimental protocols were approved by and conducted in accordance with the Ethical Committee of Experimental Animal Care, Ocean University of China.

#### 3.5.2. Cell Lines

Panc-1, HCT116, A549, HeLa, Hep G2, U2OS, U251, DU145, TE-1, BT549, MCF7, and HCT8 were from ATCC; RCC4 cell was gifted from Wuhan Xiao (Institute of Hydrobiology, Chinese Academy of Sciences) and cultured in high-glucose DMEM (Hyclone, Logan, UT, USA) with 10% FBS (PAN, Germany) and antibiotics (100 units/mL penicillin and 100 mg/mL streptomycin sulfate) at 37 °C in 5% CO_2_. The cell lines were analyzed with short tandem repeat profiling by ShCellBank (Shanghai, China). The contamination by mycoplasmas in culture cells was tested using the EZ-PCR Mycoplasmas Detection Kit (BI, Kibbutz Beit-Haemek, Israel) every three months.

#### 3.5.3. Antibody and Regent

The antibodies against Actin (abs137975) and Tubulin (abs137976) were obtained from Absin Bioscience. The antibodies against cleaved-PARP1 (13371-1-AP), cleaved-caspase-3 (40924), Cytochrome C (10993-1-AP), and BAD (10435-1-AP) were purchased from Proteintech. CCK8 and DMSO were purchased from Sigma (St. Louis, MO, USA). Enhanced chemiluminescence liquid (ECL) was from Sparkjade (Jinan, China). PVDF membranes were obtained from Millipore (Boston, MA, USA). Secondary antibodies against primary antibodies were provided from Millipore (Darmstadt, Germany). Chemical reagents were from Sinopharm (Shanghai, China).

#### 3.5.4. Cytotoxic Activity of Triphenol Derivatives

The cytotoxic activity of all the compounds, **1**–**11**, was detected using the CCK8 assay. Cells were seeded in 96-well plates at a density of approximately 4000 cells per well. After allowing the cells to adhere, they were treated with different concentrations of the derivatives for 72 h. Following the treatment period, a CCK8 solution (10%) was added to each well and incubated for an additional 2 h. The absorbance of the samples was then measured at 450 nm using a plate reader. Based on the absorbance readings, the cell viability and IC_50_ (half-maximal inhibitory concentration) values were calculated to assess the cytotoxicity of the derivatives.

#### 3.5.5. Plate Clone Formation Assay

BT549 cells were seeded in 3.5 cm dishes at a density of 200 cells per well. The cells were then treated with CHNQD-00824 at concentrations ranging between 0 and 8 μM for a period of 13 days. During this time, the growth medium was replaced every 3 days to maintain cell viability. After the treatment period, the cells were washed three times with pre-cooled phosphate-buffered saline (PBS) for 5 min each time. Subsequently, the cells were incubated with a 0.4% paraformaldehyde (Sigma, St. Louis, MO, USA) solution for 30 min. Following another three washes with pre-cooled PBS, the cells were stained with a 0.5% crystal violet solution for 30 min. The excess crystal violet (Sangon, Shanghai, China) solution was then rinsed off to remove any unbound dye. The samples were photographed and observed under an inverted microscope. Clonal populations consisting of more than 50 cells were considered as individual clones and were counted.

#### 3.5.6. Cell Proliferation Assay

BT549 cells were divided into 24-well plates with 1000 cells per well. After allowing the cells to adhere, cells were treated with CHNQD-00824 at a concentration of 5 μM. The DMSO-treated group was used as a negative control. Three wells of each treatment group were digested every 24 h and counted separately using an Automated Cell Counter (LUNA-II, logos biosystems). Counts were performed for 7 consecutive days. A cell proliferation curve was drawn to compare the cell proliferation rate.

#### 3.5.7. The Scratch Assay

Cells were seeded into 12-well plates at a density of approximately 5 × 10^5^ cells per well. Once the cells reached confluency and formed a monolayer, a 200 μL tip was used to create a scratch across the surface of the cells, ensuring an even and consistent scratch. The suspended cells were then removed by gently washing the wells with PBS solution. Next, serum-free medium containing different concentrations of CHNQD-00824 (ranging between 0 and 10 μM) was added to each well. The plates were then incubated for a specified time period, such as 0 h, 12 h, and 24 h. At each time point, the scratches were imaged using an inverted microscope. The Image-J software (version 10.0.5, Media Cybernetics Inc., Rockville, Maryland) was used to measure the area of the scratch, allowing for a quantification of cell migration. By comparing the scratch area at different time points, the rate of cell migration can be assessed.

#### 3.5.8. Cell Cycle Analysis

BT549 cells were plated in a 6 cm dish at a density of 2 × 10^6^ per well. After adherence, the cells were treated with CHNQD-00824 (0–1 μM) for 24 h. The cells were digested with pancreatic enzymes and rehung in 1.5 mL medium. Then, the cells were washed twice with pre-cooled PBS. The cells were then suspended with 70% ethanol and frozen at −20 °C overnight. After centrifugation to remove 70% ethanol, they were washed twice with pre-cooling PBS. Then, the cells were stained with a propidium iodide solution (Yeasen, Shanghai, China). The results were detected through flow cytometry and analyzed using Flowjo.

#### 3.5.9. Cell Apoptosis Assay

Approximately 2 × 10^6^ cells per well were seeded in a 6 cm dish and incubated until the cell adhered to the well. Then, the cells were treated with CHNQD-00824 (0–1 μM) for 24 h. The cells were digested with EDTA-free pancreatic enzymes and then suspended, then washed twice with pre-cooled PBS. The PBS was removed through centrifugation and the staining solution (Yeasen, Shanghai, China) containing Annexin V/FITC-PI was added. Reaction at room temperature and away from light for 10–15 min. The apoptosis was evaluated through flow cytometry and analyzed using Flowjo.

#### 3.5.10. Western Blotting Assay

BT549 cells were seeded in 6-well plates and treated with CHNQD-00824 for 24 h. Then, the cells were collected and lysed with loading buffer. The protein lysates were then separated using SDS-PAGE (Sangon, Shanghai, China) and transferred into nitrocellulose membranes (Millipore, Billerica, MA, USA). To prevent non-specific binding, the membranes were blocked with skim milk powder for 2 h. Then, membranes were incubated overnight with specific primary antibodies at 4 °C and secondary antibodies at room temperature for 2 h. The enhanced chemiluminescence kit was then prepared and added to the membranes in drops, and photographed and analyzed using a protein imager (Universal Hood II, BIO-RAD, Milan, Italy).

#### 3.5.11. AO Staining Assay

Zebrafish embryos were obtained by mating wild-type female and male zebrafish. The embryos were then cultured in embryo medium containing PTU until they reached 24 h post-fertilization (hpf). After the 24 h culture period, the embryos were treated with varying concentrations of CHNQD-00824, ranging from 0 to 15 μM, for an additional 24 h. Following the treatment, the embryos were stained with acridine orange (AO) at a concentration of 2 μg/mL for 30 min. The stained embryos were then washed three times with embryo culture medium to remove any excess dye. The results were visualized and photographed using a fluorescence microscope (SMZ645, Nikon, Tokyo, Japan). The Image-pro-plus software (version 10.0.5, Media Cybernetics Inc., Rockville, Maryland) was used to analyze the images and quantify the results obtained from the stained embryos.

#### 3.5.12. In Vivo Anticancer Assay of Zebrafish

*To*(*kras^G12V^*) genotype female and male zebrafish were mated to obtain embryos. Embryos were cultured to 3 dpf in embryo medium containing PTU and then embryos were treated with CHNQD-00824 (0–5 μM) and DOX (60 mg/L, Sangon, China) for 4 days in embryo medium containing PTU. The embryo culture medium was changed once at 5 dpf. At 7 dpf, zebrafish were anesthetized with tracaine (0.08%, *w*/*v*) and transferred to methyl-cellulose (2%, *w*/*v*) for photo observation. The results were photographed using a fluorescence microscope and analyzed using image-pro-plus.

#### 3.5.13. Statistics

The Prism 8 software was used for statistical analysis. The results were shown as the mean ± SD. For all data, *p*-value < 0.05 was considered statistically significant.

## 4. Conclusions

The findings of this study highlight the discovery of a terphenyllin derivative called CHNQD-00824 from the marine compound library. CHNQD-00824 has shown potential as an anticancer agent. Moreover, further investigations revealed that CHNQD-00824 has the ability to induce DNA damage. DNA damage is a crucial mechanism in cancer treatment as it can lead to cell death or inhibit cell proliferation. This finding suggests that CHNQD-00824 may be acting through a mechanism that disrupts the integrity of cancer cell DNA. In addition to its activity against multiple cell lines in vitro, CHNQD-00824 was evaluated in a DOX-induced liver-specific enlargement model in zebrafish. In this model, CHNQD-00824 significantly suppressed tumor growth when administered at a concentration of 5 μM. Zebrafish have emerged as a valuable model organism in cancer research, complementing the insights gained from murine models and cell culture systems. Their small size, rapid development, genetic conservation, and ease of genome manipulation make them an excellent tool for studying tumor initiation, progression, and response to treatment. This observation suggests that CHNQD-00824 may have potential in inhibiting tumor growth in vivo, making it a promising candidate for further development as a cancer drug agent. Further research is needed to explore the mechanisms, pharmacokinetics, and safety profile of CHNQD-00824 in order to assess its clinical potential.

## Figures and Tables

**Table 1:** Cytotoxic activity of CHNQD-00824 against cancer cell lines.

Cell Lines	RCC4	HeLa	Hep G2	U2OS	U251	DU145	TE-1	BT549	HCT8	MCF7
IC_50_ (μM)	2.65 ± 0.04	0.45 ± 0.03	7.64 ± 0.21	0.44 ± 0.07	5.43 ± 0.05	0.91 ± 0.04	4.35 ± 0.12	0.16 ±0.02	0.61 ± 0.06	3.66 ± 0.37

## Data Availability

The data are contained within the article or Supplementary Material.

## Supplementary Materials

The following supporting information can be downloaded at: https://www.mdpi.com/article/10.3390/md21100512/s1, Figure S1–S33: 1H NMR, 13C NMR. Figure S34: Fermentation yield comparison chart. Figure S35: UPLC analysis of the reaction process of terphenyllin **1** treated with allyl bromide (2.5 equivalent).

## References

[1] Siegel R.L., Miller K.D., Jemal A. (2020). Cancer statistics, 2020. CA Cancer J. Clin..

[2] Liang W.H., Guan W.J., Chen R.C., Wang W., Li J.F., Xu K., Li C.C., Ai Q., Lu W.X., Liang H.R. (2020). Cancer patients in SARS-CoV-2 infection: A nationwide analysis in China. Lancet Oncol..

[3] Siegel R.L., Miller K.D., Wagle N.S., Jemal A. (2023). Cancer statistics, 2023. CA Cancer J. Clin..

[4] Newman D.J., Cragg G.M. (2020). Natural Products as Sources of New Drugs over the Nearly Four Decades from 01/1981 to 09/2019. J. Nat. Prod..

[5] Carroll A.R., Copp B.R., Davis R.A., Keyzers R.A., Prinsep M.R. (2022). Marine natural products. Nat. Prod. Rep..

[6] Carroll A.R., Copp B.R., Davis R.A., Keyzers R.A., Prinsep M.R. (2023). Marine natural products. Nat. Prod. Rep..

[7] Haque N., Parveen S., Tang T.T., Wei J.E., Huang Z.N. (2022). Marine Natural Products in Clinical Use. Mar. Drugs.

[8] Wang C.F., Ma J., Jing Q.Q., Cao X.Z., Chen L., Chao R., Zheng J.Y., Shao C.L., He X.X., Wei M.Y. (2022). Integrating Activity-Guided Strategy and Fingerprint Analysis to Target Potent Cytotoxic Brefeldin A from a Fungal Library of the Medicinal Mangrove *Acanthus ilicifolius*. Mar. Drugs..

[9] Lu X.X., Jiang Y.Y., Wu Y.W., Chen G.Y., Shao C.L., Gu Y.C., Liu M., Wei M.Y. (2022). Semi-Synthesis, Cytotoxic Evaluation, and Structure—Activity Relationships of Brefeldin A Derivatives with Antileukemia Activity. Mar. Drugs..

[10] Ren X.H., Xie X.Y., Chen B.X., Liu L., Jiang C.Q., Qian Q. (2021). Marine Natural Products: A Potential Source of Anti-hepatocellular Carcinoma Drugs. J. Med. Chem..

[11] Lu W.Y., Li H.J., Li Q.Y., Wu Y.C. (2021). Application of marine natural products in drug research. Bioorgan. Med. Chem..

[12] Guo F.W., Zhang Q., Gu Y.C., Shao C.L. (2023). Sulfur-containing marine natural products as leads for drug discovery and development. Curr. Opin. Chem. Biol..

[13] Holland D.C., Carroll A.R. (2023). Marine indole alkaloid diversity and bioactivity. What do we know and what are we missing?. Nat. Prod. Rep..

[14] Maha Z.F., Hurley L.H. (1999). Ecteinascidin 743: A minor groove alkylator that bends DNA toward the major groove. J. Med. Chem..

[15] Uemura D., Takahashi K., Yamamoto T. (1985). Norhalichondrin A: An antitumor polyether macrolide from a marine sponge. J. Am. Chem. Soc..

[16] Mayer A.M.S., Glaser K.B., Cuevas C., Jacobs R.S., Kem W., Little R.D., McIntosh J.M., Newman D.J., Potts B.C., Shuster D.E. (2010). The odyssey of marine pharmaceuticals: A current pipeline perspective. Trends Pharmacol. Sci..

[17] Francisco J.A., Cerveny C.G., Meyer D.L., Mixan B.J., Klussman K., Chace D.F., Rejniak S.X., Gordon K.A., DeBlanc R., Toki B.E. (2003). cAC10-vcMMAE, an anti-CD30-monomethyl auristatin E conjugate with potent and selective antitumor activity. Blood.

[18] Tai Y.T., Mayes P.A., Acharya C., Zhong M.Y., Cea M., Cagnetta A., Craigen J., Yates J., Gliddon L., Fieles W. (2014). Novel anti-B-cell maturation antigen antibody-drug conjugate (GSK2857916) selectively induces killing of multiple myeloma. Blood.

[19] Hai Y., Wei M.Y., Wang C.Y., Gu Y.C., Shao C.L. (2021). The intriguing chemistry and biology of sulfur-containing natural products from marine microorganisms (1987–2020). Mar. Life Sci. Technol..

[20] Hai Y., Cai Z.M., Li P.D., Wei M.Y., Wang C.Y., Gu Y.C., Shao C.L. (2022). Trends of antimalarial marine natural products: Progresses, challenges and opportunities. Nat. Prod. Rep..

[21] Xu W.F., Wu N.N., Wu Y.W., Qi Y.X., Wei M.Y., Pineda L.M., Ng M.G., Spadafora C., Zheng J.Y., Lu L. (2022). Structure modification, antialgal, antiplasmodial, and toxic evaluations of a series of new marine-derived 14-membered resorcylic acid lactone derivatives. Mar. Life Sci. Technol..

[22] Jiang Y.Y., Gao Y., Liu J.Y., Xu Y., Wei M.Y., Wang C.Y., Gu Y.C., Shao C.L. (2022). Design and Characterization of a Natural Arf-GEFs Inhibitor Prodrug CHNQD-01255 with Potent Anti-Hepatocellular Carcinoma Efficacy in Vivo. J. Med. Chem..

[23] Chen J., Xu L., Zhang X.Q., Liu X., Zhang Z.X., Zhu Q.M., Liu J.Y., Iqbal M.O., Ding N., Shao C.L. (2023). Discovery of a natural small-molecule AMP-activated kinase activator that alleviates nonalcoholic steatohepatitis. Mar. Life Sci. Technol..

[24] Zhang X.Q., Mou X.F., Mao N., Hao J.J., Liu M., Zheng J.Y., Wang C.Y., Gu Y.C., Shao C.L. (2018). Design, semisynthesis, α-glucosidase inhibitory, cytotoxic, and antibacterial activities of p-terphenyl derivatives. Eur. J. Med. Chem..

[25] Haider W., Xu W.F., Liu M., Wu Y.W., Tang Y.F., Wei M.Y., Wang C.Y., Lu L., Shao C.L. (2020). Structure-Activity Relationships and Potent Cytotoxic Activities of Terphenyllin Derivatives from a Small Compound Library. Chem. Biodivers..

[26] Bailly C. (2022). Anti-inflammatory and anticancer p-terphenyl derivatives from fungi of the genus *Thelephora*. Bioorgan. Med. Chem..

[27] Song Y.J., Zheng H.B., Peng A.H., Ma J.H., Lu D.D., Li X., Zhang H.Y., Xie W.D. (2019). Strepantibins A–C: Hexokinase II inhibitors from a mud dauber wasp associated *Streptomyces* sp.. J. Nat. Prod..

[28] Liu J.K. (2006). Natural terphenyls: Developments since 1877. Chem. Rev..

[29] Feng D.Q., Biftu T., Romero F.A., Kekec A., Dropinski J., Kassick A., Xu S.Y., Kurtz M.M., Gollapudi A., Shao Q. (2018). Discovery of MK-8722: A systemic, direct pan-activator of AMP-activated protein kinase. Med. Chem. Lett..

[30] Marchelli R., Vining L.C. (1975). Terphenyllin, a novel p-terphenyl metabolite from *Aspergillus candidus*. J. Antibiot..

[31] Kurobane I., Vining L.C., Mcinnes A.G., Smith D.G. (1979). 3-Hydroxyterphenyllin, a new metabolite of *Aspergillus candidus*. J. Antibio..

[32] Wei H., Inada H., Hayashi A., Higashimoto K., Pruksakorn P., Kamada S., Arai M., Ishida S., Kobayashi M. (2007). Prenylterphenyllin and its dehydroxyl analogs, new cytotoxic substances from a marine-derived fungus *Aspergillus candidus* IF10. J. Antibiot..

[33] Takahashi C., Yoshihira K., Natori S., Umeda M., Ohtsubo K., Saito M. (1974). Toxic metabolites of *Aspergillus candidus*. Cell. Mol. Life Sci..

[34] Takahashi C., Yoshihira K., Natori S., Umeda M. (1976). The structures of toxic metabolites of *Aspergillus candidus*. I. The compounds A and E, cytotoxic *p*-terphenyls. Chem. Pharm. Bull..

[35] Brix N., Samaga D., Belka C., Zitzelsberger H., Lauber K. (2021). Analysis of clonogenic growth in vitro. Nat. Protoc..

[36] Lambert A.W., Pattabiraman D.R., Weinberg R.A. (2017). Emerging Biological Principles of Metastasis. Cell.

[37] Welch D.R., Hurst D.R. (2019). Defining the Hallmarks of Metastasis. Cancer Res..

[38] Martinotti S., Ranzato E. (2020). Scratch Wound Healing Assay. Methods Mol. Biol..

[39] Nagata S. (2018). Apoptosis and Clearance of Apoptotic Cells. Annu. Rev. Immunol..

[40] Brown J.S., O’Carrigan B., Jackson S.P., Yap T.A. (2017). Targeting DNA Repair in Cancer: Beyond PARP Inhibitors. Cancer Discov..

[41] Bui N.L., Pandey V., Zhu T., Ma L., Basappa P.E.L. (2018). Bad phosphorylation as a target of inhibition in oncology. Cancer Lett..

[42] Kalpage H.A., Bazylianska V., Recanati M.A., Fite A., Liu J., Wan J., Mantena N., Malek M.H., Podgorski I., Heath E.I. (2019). Tissue-specific regulation of cytochrome c by post-translational modifications: Respiration, the mitochondrial membrane potential, ROS, and apoptosis. FASEB J..

[43] Sharma A., Singh K., Almasan A. (2012). Histone H2AX phosphorylation: A marker for DNA damage. Methods Mol. Biol..

[44] Eimon P.M., Ashkenazi A. (2010). The zebrafish as a model organism for the study of apoptosis. Apoptosis.

[45] Santoriello C., Zon L.I. (2012). Hooked! Modeling human disease in zebrafish. J. Clin. Investig..

[46] Langheinrich U. (2003). Zebrafish: A new model on the pharmaceutical catwalk. BioEssays.

[47] Letrado P., Miguel I.D., Lamberto1 I., Díez-Martínez R., Oyarzabal J. (2018). Zebrafish: Speeding Up the Cancer Drug Discovery Process. Cancer Res..

[48] Loibl S., Poortmans P., Morrow M., Denkert C., Curigliano G. (2021). Breast cancer. Lancet.

[49] Wang S., Li X.M., Teuscher F., Li D.L., Diesel A., Ebel R., Proksch P., Wang B.G. (2006). Chaetopyranin, a benzaldehyde derivative, and other related metabolites from Chaetomium globosum, an endophytic fungus derived from the marine red alga Poly-siphonia urceolata. J. Nat. Prod..

