# Supplementary material for: A Terphenyllin Derivative CHNQD-00824 from the Marine Compound Library Induced DNA Damage as a Potential Anticancer Agent
Source: Mar Drugs. 2023 Sep 27;21(10):512. doi: 10.3390/md21100512 (PMC10608154; doi:10.3390/md21100512)
Supplement: Supplementary file 1 [file marinedrugs-21-00512-s001.zip › marinedrugs-2633877-supplementary.pdf]

A Terphenyllin Derivative CHNQD-00824 from the Marine Compound Library Induced DNA Damage as a Potential Anticancer Agent

Xi-Zhen Cao [1,2,†], Bo-Qi Zhang [1,2,†], Cui-Fang Wang [1,2], Jun-Na Yin [1,2], Waqas Haider [1,2] ,

Gulab Said [1,3] , Mei-Yan Wei [1,2] and Ling Lu [1,2,*]

[1] Key Laboratory of Marine Drugs, The Ministry of Education of China, School of Medicine and Pharmacy, Ocean University of China, Qingdao 266003, China

[2] Laboratory for Marine Drugs and Biological Products, Laoshan Laboratory, Qingdao 266003, China
[3] Department of Chemistry, Women University Swabi, Swabi 23430, Pakistan
[*] Correspondence: linglu@ouc.edu.cn
[†] These authors contributed equally to this work.

Content of Supporting Information

NMR data for natural products **1-4** and derivatives **7-11**.

Figure S1. ^{1}H NMR (400 MHz, DMSO-d_6) spectrum of natural compound **1**.

Figure S2. ^{13}C NMR (100 MHz, DMSO-d_6) spectrum of natural compound **1**.

Figure S3. ESIMS spectrum of natural compound **1**.

Figure S4. ^{1}H NMR (400 MHz, DMSO-d_6) spectrum of natural compound **2**.

Figure S5. ^{13}C NMR (100 MHz, DMSO-d_6) spectrum of natural compound **2**.

Figure S6. ESIMS spectrum of natural compound **2**.

Figure S7. ^{1}H NMR (400 MHz, Acetone-d_6) spectrum of natural compound **3**.

Figure S8. ^{13}C NMR (100 MHz, Acetone-d_6) spectrum of natural compound **3**.

Figure S9. ESIMS spectrum of natural compound **3**.

Figure S10. ^{1}H NMR (400 MHz, DMSO-d_6) spectrum of natural compound **4**.

Figure S11. ^{13}C NMR (100 MHz, DMSO-d_6) spectrum of natural compound **4**.

Figure S12. ESIMS spectrum of natural compound **4**.

Figure S13. ^{1}H NMR (400 MHz, Acetone-d_6) spectrum of terphenyllin derivative compound **5**.

Figure S14. ^{13}C NMR (100 MHz, Acetone-d_6) spectrum of terphenyllin derivative compound **5**.

Figure S15. HRESIMS spectrum of terphenyllin derivative compound **5.**

Figure S16. ^{1}H NMR (400 MHz, Acetone-d_6) spectrum of terphenyllin derivative compound **6**.

Figure S17. ^{13}C NMR (100 MHz, Acetone-d_6) spectrum of terphenyllin derivative compound **6**.

Figure S18. HRESIMS spectrum of terphenyllin terphenyllin derivative compound **6.**

Figure S19. ^{1}H NMR (400 MHz, DMSO-d_6) spectrum of terphenyllin derivative compound **7**.

Figure S20. ^{13}C NMR (100 MHz, DMSO-d_6) spectrum of terphenyllin derivative compound **7**.

Figure S21. ESIMS spectrum of terphenyllin derivative compound **7**.

Figure S22. ^{1}H NMR (400 MHz, $CDCl_3$) spectrum of terphenyllin derivative compound **8**.

Figure S23. ^{13}C NMR (100 MHz, $CDCl_3$) spectrum of terphenyllin derivative compound **8**.

Figure S24. ESIMS spectrum of terphenyllin derivative compound **8**.

Figure S25. ^{1}H NMR (400 MHz, DMSO-d_6) spectrum of terphenyllin derivative compound **9**.

Figure S26. ^{13}C NMR (100 MHz, DMSO-d_6) spectrum of terphenyllin derivative compound **9**.

Figure S27. ESIMS spectrum of terphenyllin derivative compound **9**.

Figure S28. ^{1}H NMR (400 MHz, DMSO-d_6) spectrum of terphenyllin derivative compound **10**.

Figure S29. ^{13}C NMR (100 MHz, DMSO-d_6) spectrum of terphenyllin derivative compound **10**.

Figure S30. ESIMS spectrum of terphenyllin derivative compound **10**.

Figure S31. ^{1}H NMR (400 MHz, DMSO-d_6) spectrum of terphenyllin derivative compound **11**.

Figure S32. ^{13}C NMR (100 MHz, DMSO-d_6) spectrum of terphenyllin derivative compound **11**.

Figure S33. ESIMS spectrum of terphenyllin derivative compound **11**.

Figure S34. Fermentation yield comparison chart.

Figure S35. UPLC analysis of the reaction process of terphenyllin **1** treated with allyl bromide (2.5 equivalent).

Terphenyllin (**1**), White, amorphous powder; ^{1}H NMR (400 MHz, DMSO-d_6) δ 7.44 (2H, d, J = 8.6 Hz), 7.10 (2H, d, J = 8.6 Hz), 6.84 (2H, d, J = 8.6 Hz), 6.76 (2H, d, J = 8.6 Hz), 6.39 (1H, s), 3.64 (3H, s), 3.30 (3H, s).^{13}C NMR (100 MHz, DMSO-d_6) δ 156.8 (C), 155.9 (C), 153.1 (C), 148.2 (C), 139.3 (C), 132.4 (C), 131.9 (CH×2), 129.7 (CH×2), 128.7 (C), 124.5 (C), 116.9 (C), 115.2 (CH×2), 114.3 (CH×2), 102.9 (CH), 60.1 (CH_3), 55.6 (CH_3). ESIMS m/z 339.05 [M + H]$^{+}$.

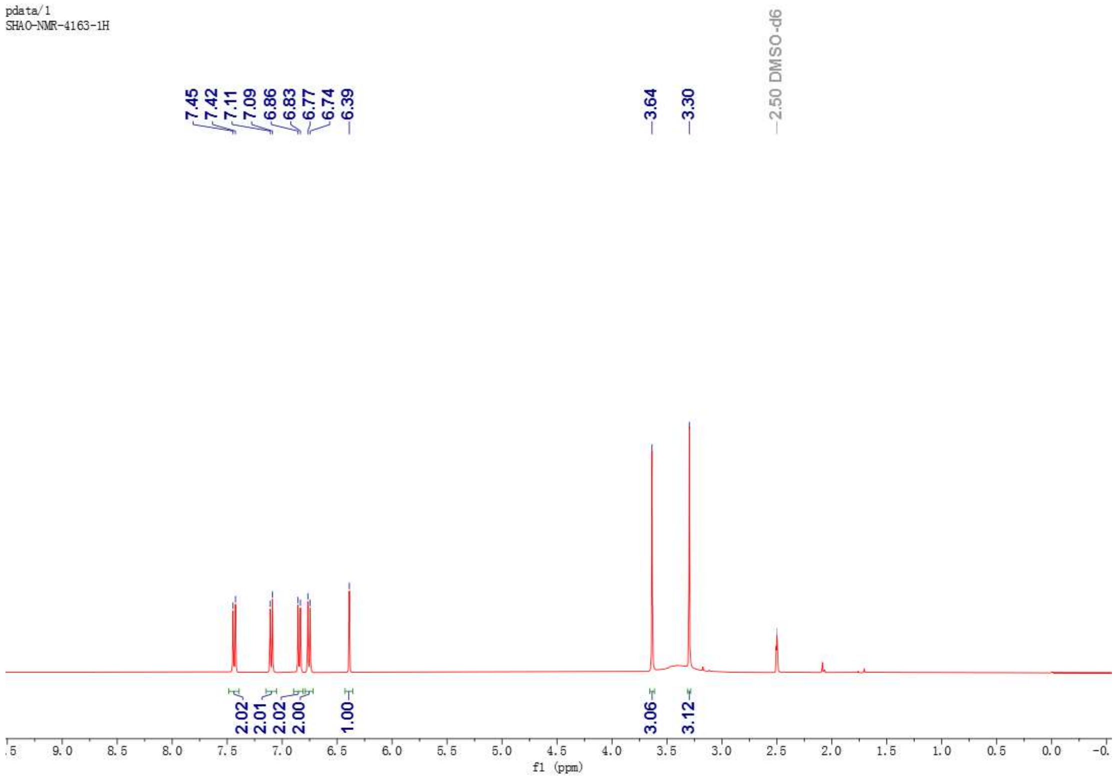

Figure S1. ^{1}H NMR (400 MHz, DMSO-d_6) spectrum of natural compound **1**.

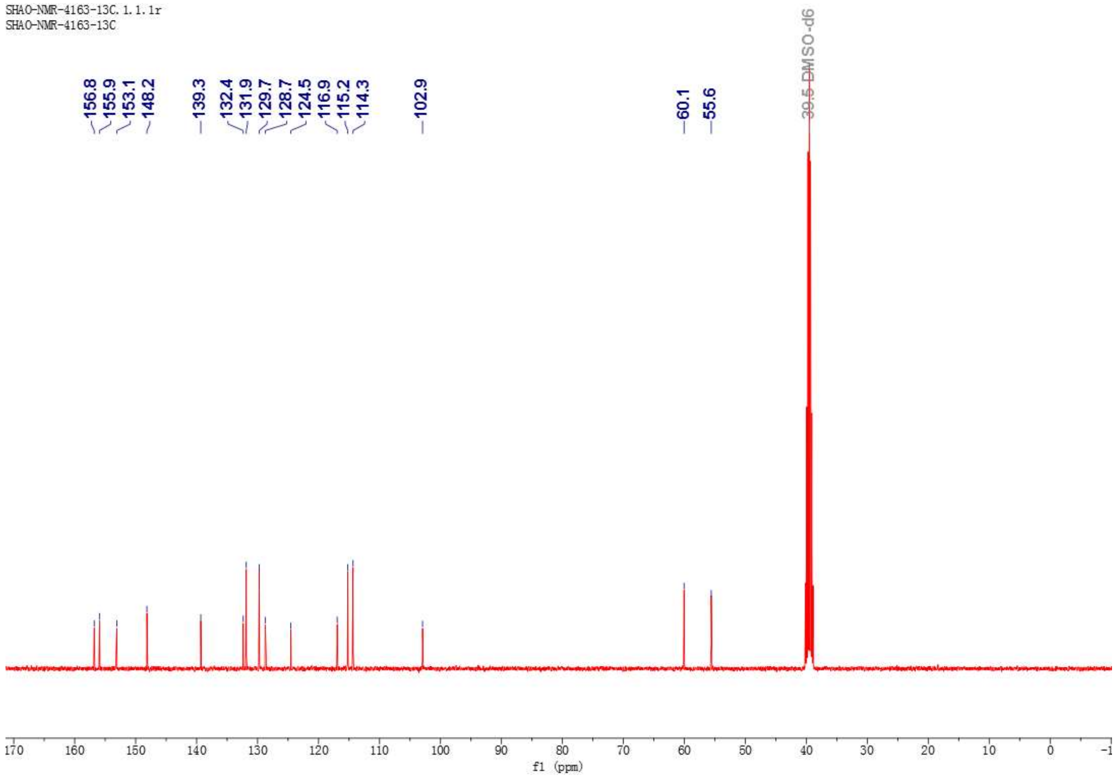

Figure S2. ^{13}C NMR (100 MHz, DMSO-d_6) spectrum of natural compound **1**.

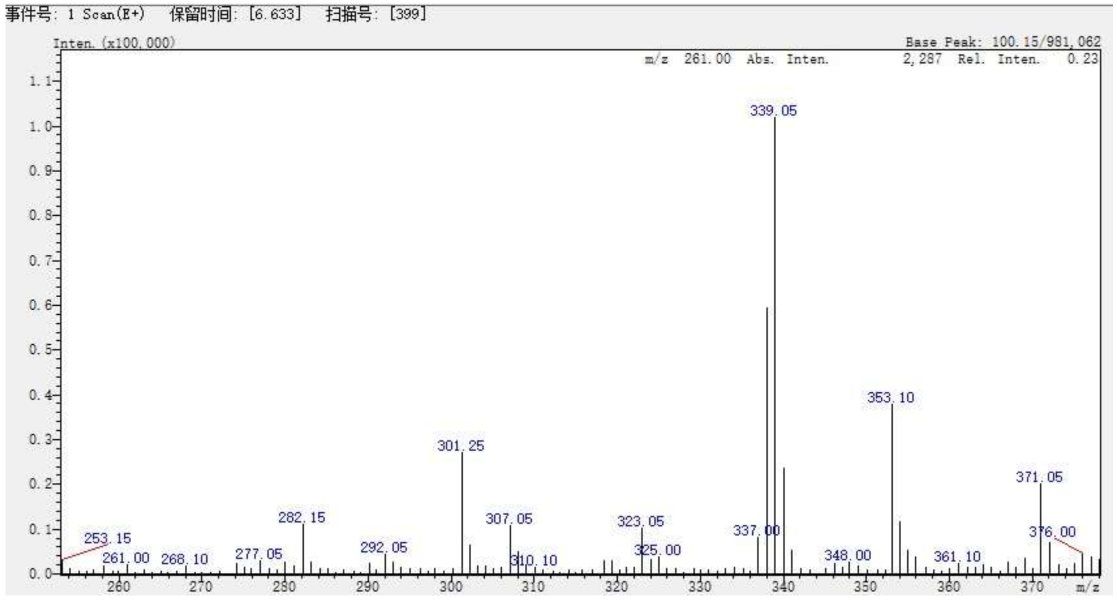

Figure S3. ESIMS spectrum of natural compound **1**.

3-hydroxyterphenyllin (**2**), White, amorphous powder; ^{1}H NMR (400 MHz, DMSO-d_6) δ 7.43 (2H, d, J = 8.7 Hz), 6.84 (2H, d, J = 8.7 Hz), 6.74 – 6.67 (2H, m), 6.54 (1H, dd, J = 8.1, 2.0 Hz), 6.37 (1H, s), 3.63 (3H, s), 3.30 (3H, s).^{13}C NMR (100 MHz, DMSO-d_6) δ 156.8 (C), 153.2 (C), 148.2 (C), 144.4 (C), 144.1 (C), 139.3 (C), 132.3 (C), 129.8 (CH×2), 128.8 (C), 125.0 (C), 122.0 (CH), 118.5 (CH), 117.3 (CH), 115.3 (CH×2), 114.9 (C), 103.0 (CH), 60.1 (CH_3),55.7 (CH_3). ESIMS m/z 355.05 $[M + H]^+$.

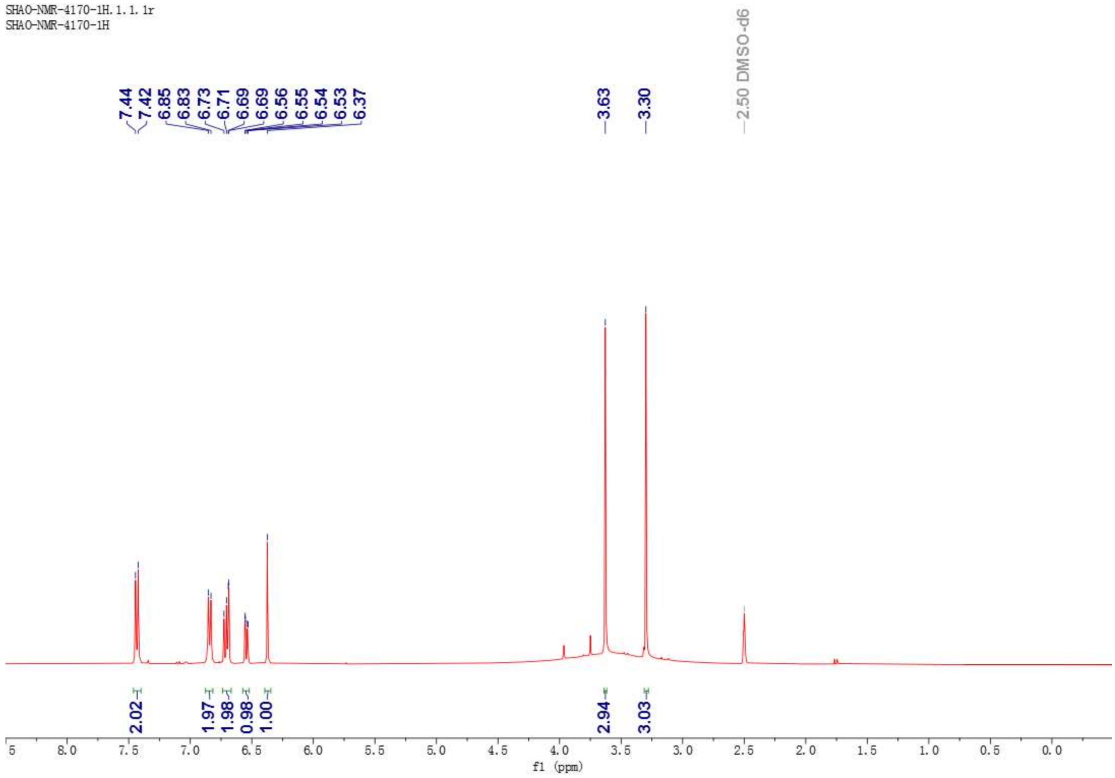

Figure S4. ^{1}H NMR (400 MHz, DMSO-d_6) spectrum of natural compound **2**.

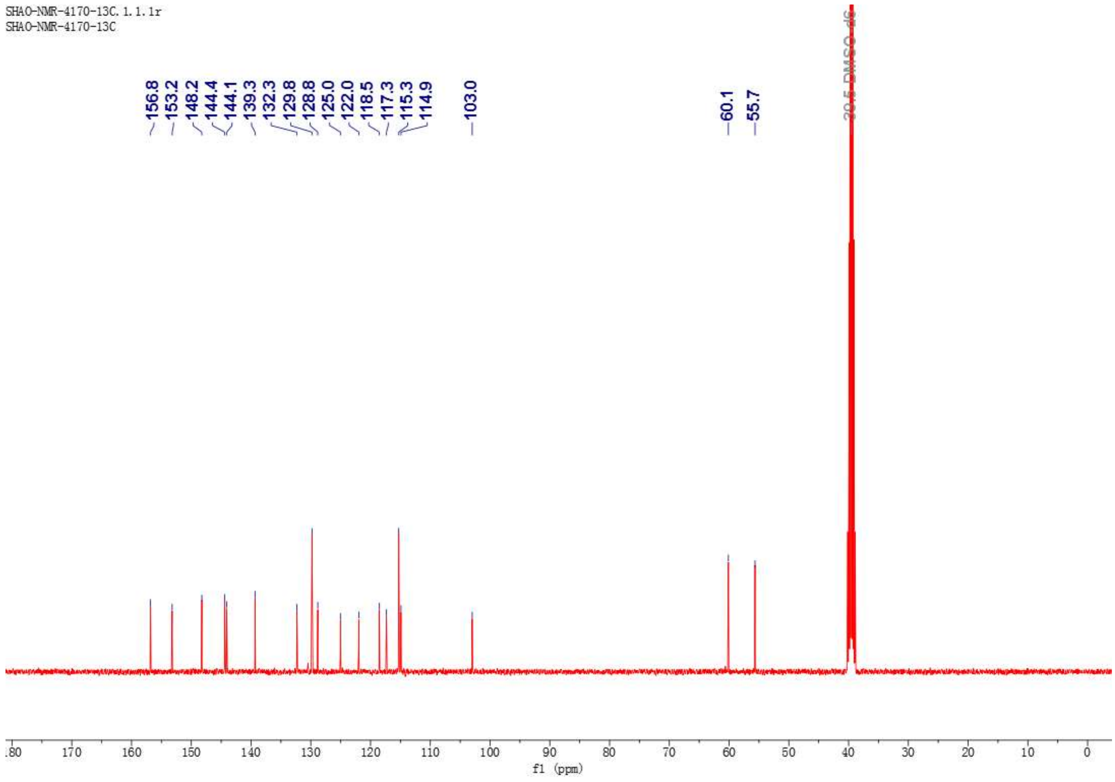

Figure S5. ^{13}C NMR (100 MHz, DMSO-d_6) spectrum of natural compound **2**.

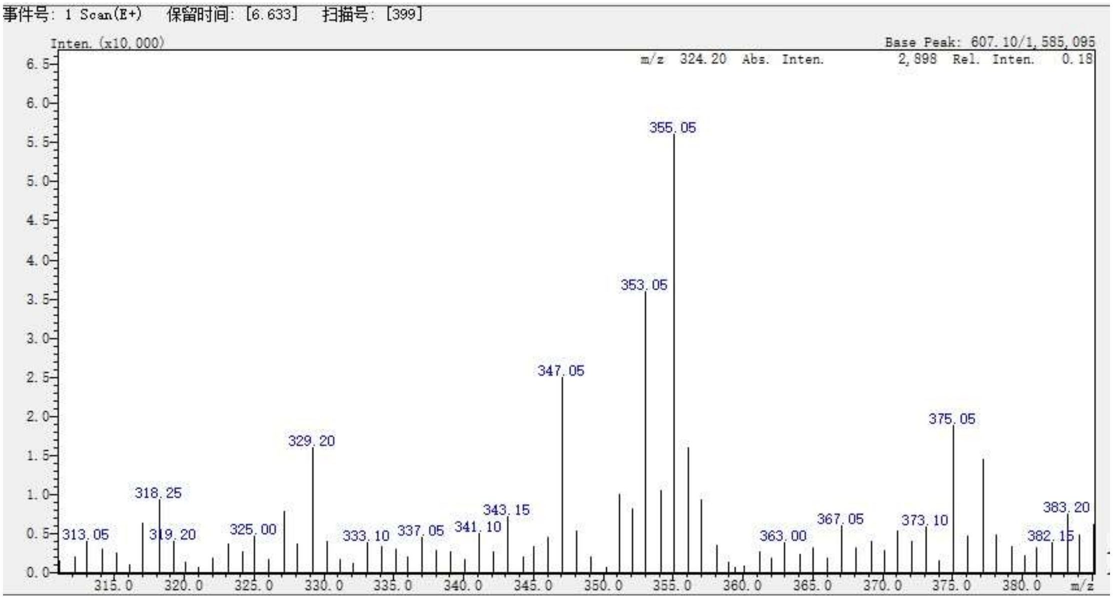

Figure S6. ESIMS spectrum of natural compound **2**.

Prenylterphenyllin (**3**), White, amorphous powder; ^{1}H NMR (400 MHz, Acetone-d_6) δ 7.66 (2H, d, J = 6.9 Hz), 7.46 (2H, t, J = 7.4 Hz), 7.37 (1H, t, J = 7.4 Hz), 6.96 (1H, d, J = 8.3 Hz), 6.92 (1H, d, J = 2.0 Hz), 6.83 (1H, dd, J= 8.3, 2.0 Hz), 6.52 (1H, s), 5.77 – 5.38 (1H, m), 4.63 (2H, dt, J = 6.5, 1.1 Hz), 3.73 (3H, s), 3.38 (3H, s), 2.26 (1H, s), 1.83 – 1.73 (6H, m).^{13}C NMR (100 MHz, Acetone-d_6) δ 154.6 (C), 149.3 (C), 147.0 (C), 146.3 (C), 140.3 (C), 139.6 (C), 137.5 (CH), 133.8 (C), 129.8 (CH×2), 129.2 (CH×2), 128.1 (C), 127.9 (CH), 123.2 (CH), 121.5 (CH), 119.0 (CH), 118.7 (C), 113.1 (C), 104.4 (CH), 66.4 (CH3), 60.9 (CH3), 56.2 (CH2), 25.9 (CH3), 18.3 (CH3). ESIMS m/z 407.15 [M+H]$^+$.

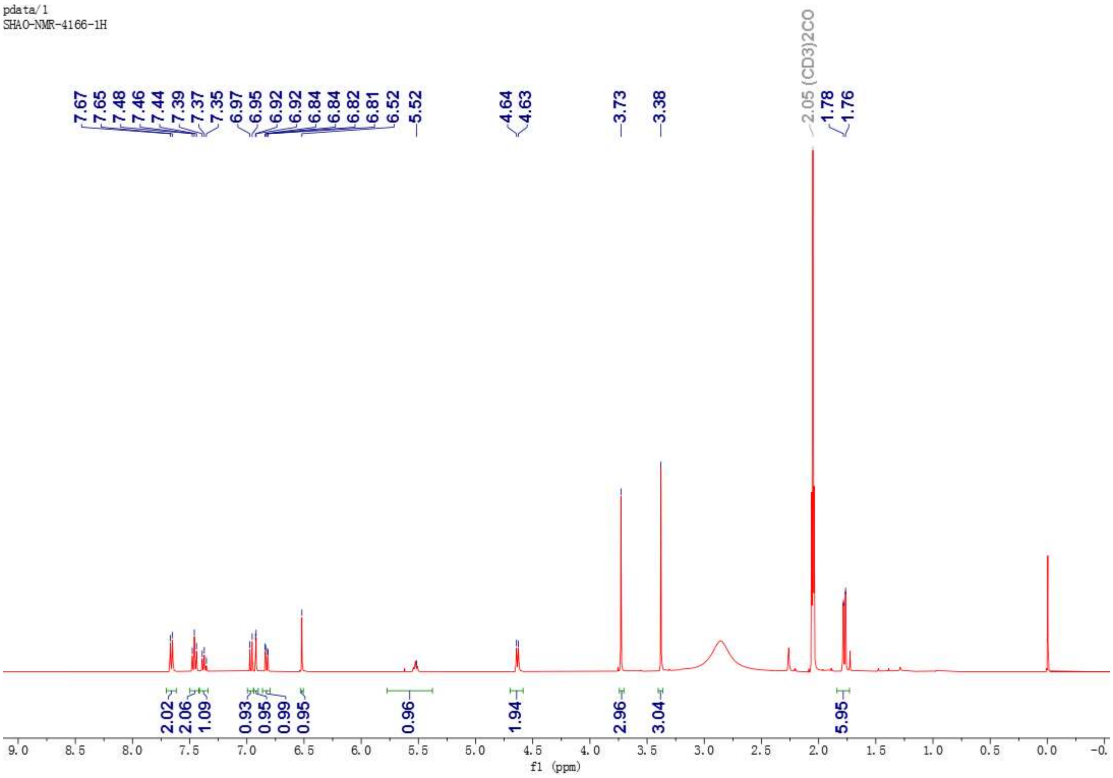

Figure S7. ^{1}H NMR (400 MHz, Acetone-d_6) spectrum of natural compound **3**.

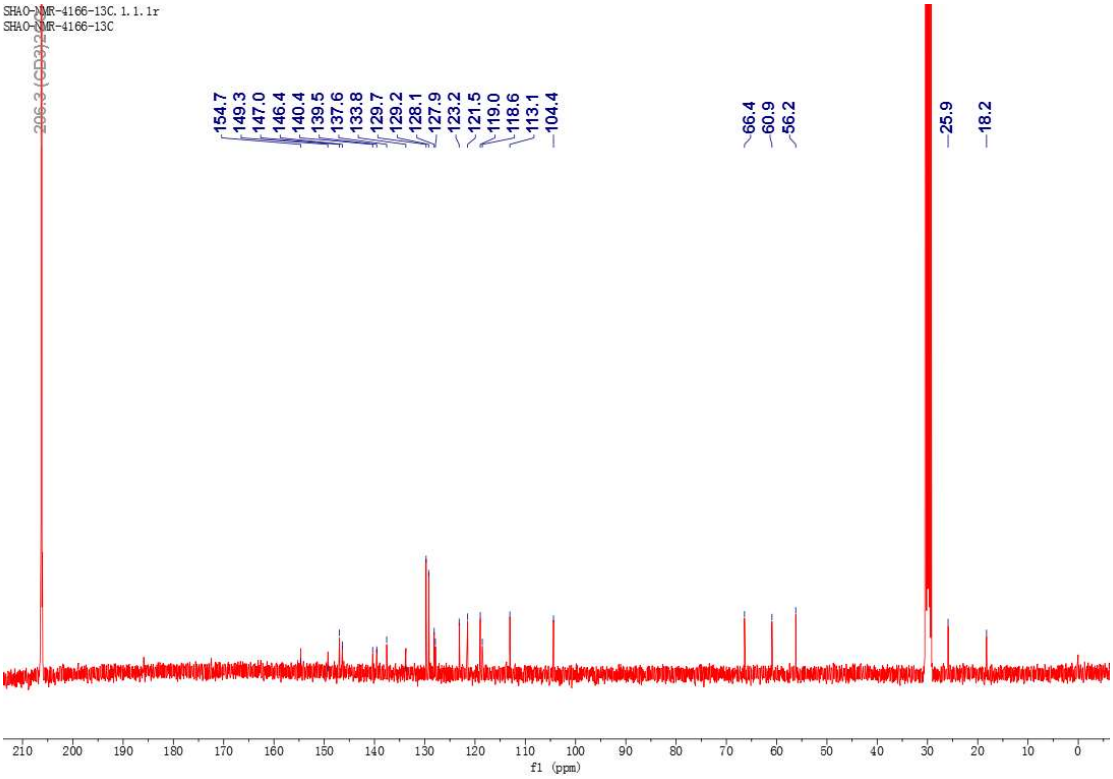

Figure S8. ^{13}C NMR (100 MHz, Acetone-d_6) spectrum of natural compound **3**.

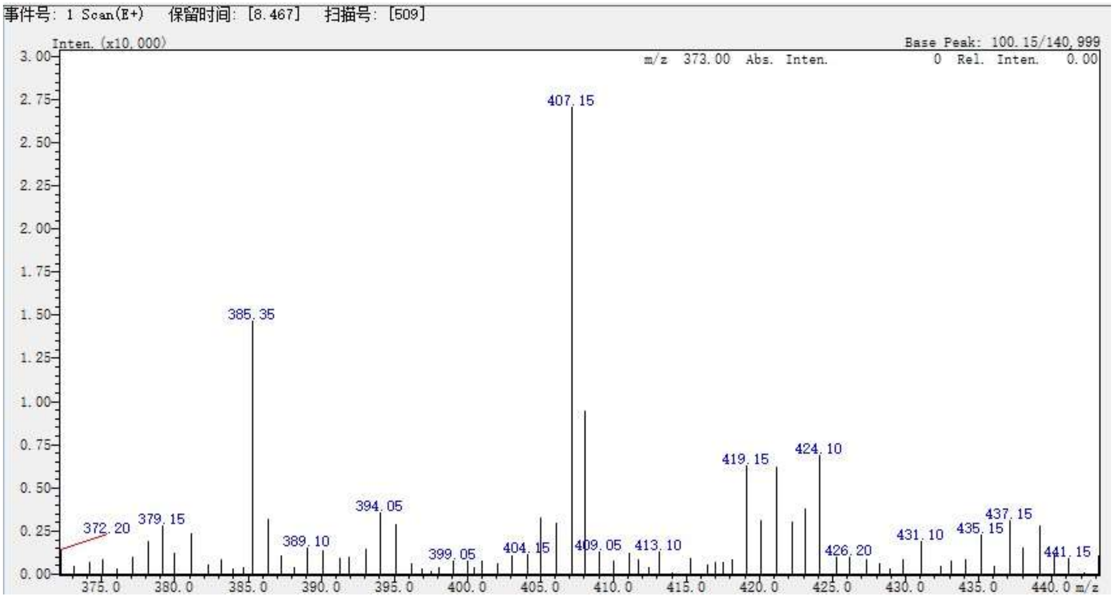

Figure S9. ESIMS spectrum of natural compound **3**.

Deoxyterhenyllin (**4**), White, amorphous powder; ^{1}H NMR (400 MHz, DMSO-d_6) δ 9.32 (1H, s), 8.60 (1H, s), 7.69 – 7.52 (2H, m), 7.46 (2H, t, *J* = 7.4 Hz), 7.37 (1H, t, *J* = 7.4 Hz), 7.17 – 7.01 (2H, m), 6.82 – 6.69 (2H, m), 6.44 (1H, s), 3.65 (3H, s), 3.30 (3H, s).^{13}C NMR (100 MHz, DMSO-d_6) δ 156.0 (C), 153.2 (C), 148.2 (C), 139.5 (C), 138.2 (C), 132.4 (C), 131.8 (CH×2), 128.7 (CH×2), 128.3 (CH×2), 127.2 (CH), 124.4 (C), 117.8 (C), 114.4 (CH×2), 103.2 (CH), 60.3 (CH_3), 55.6 (CH_3). ESIMS m/z 323.10 $[M + H]^+$.

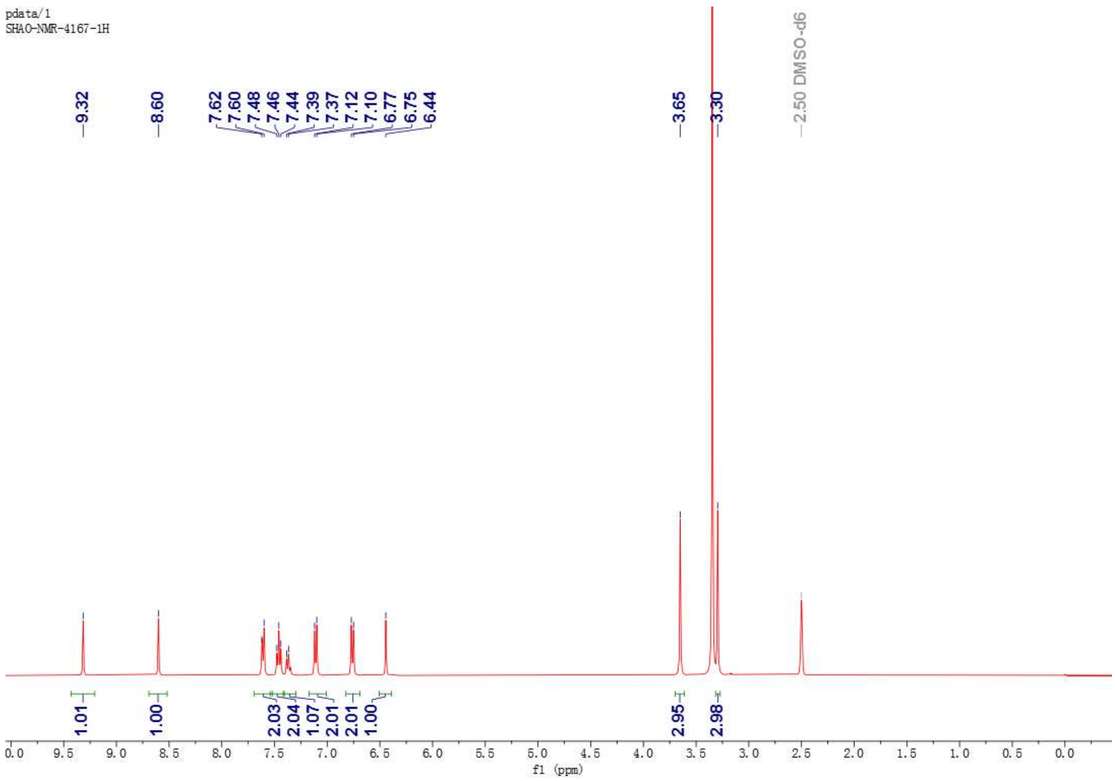

Figure S10. ^{1}H NMR (400 MHz, DMSO-d_6) spectrum of natural compound **4**.

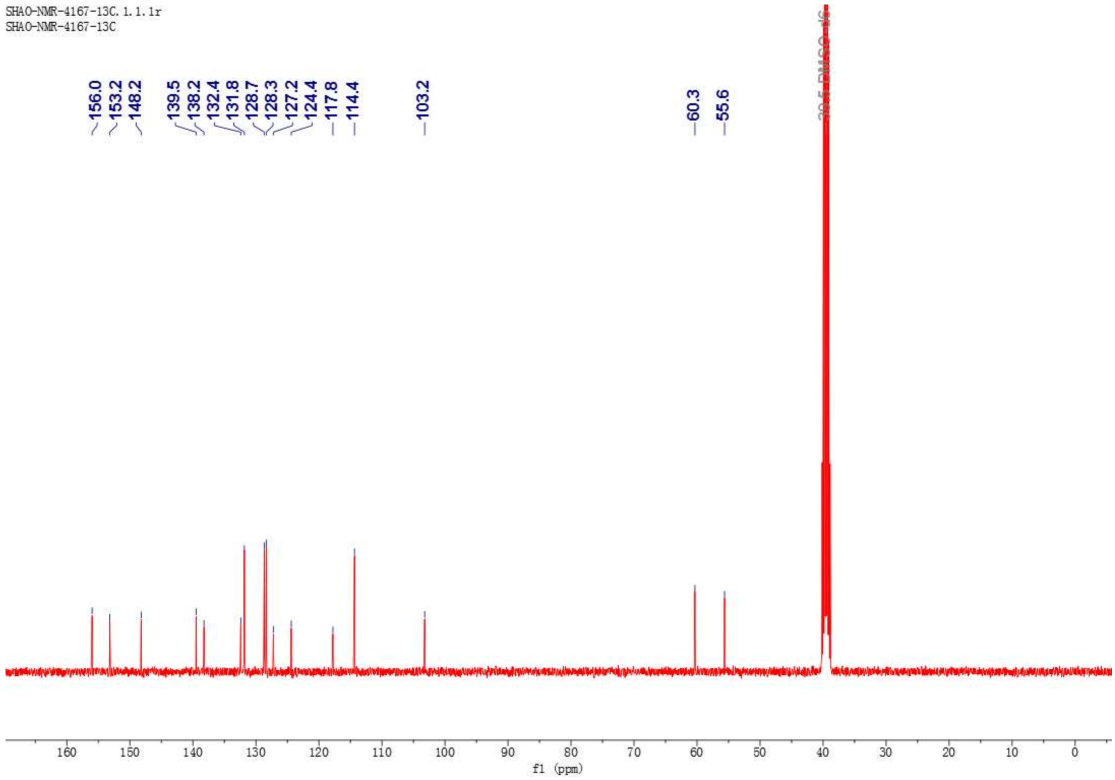

Figure S11. ^{13}C NMR (100 MHz, DMSO-d_6) spectrum of natural compound **4**.

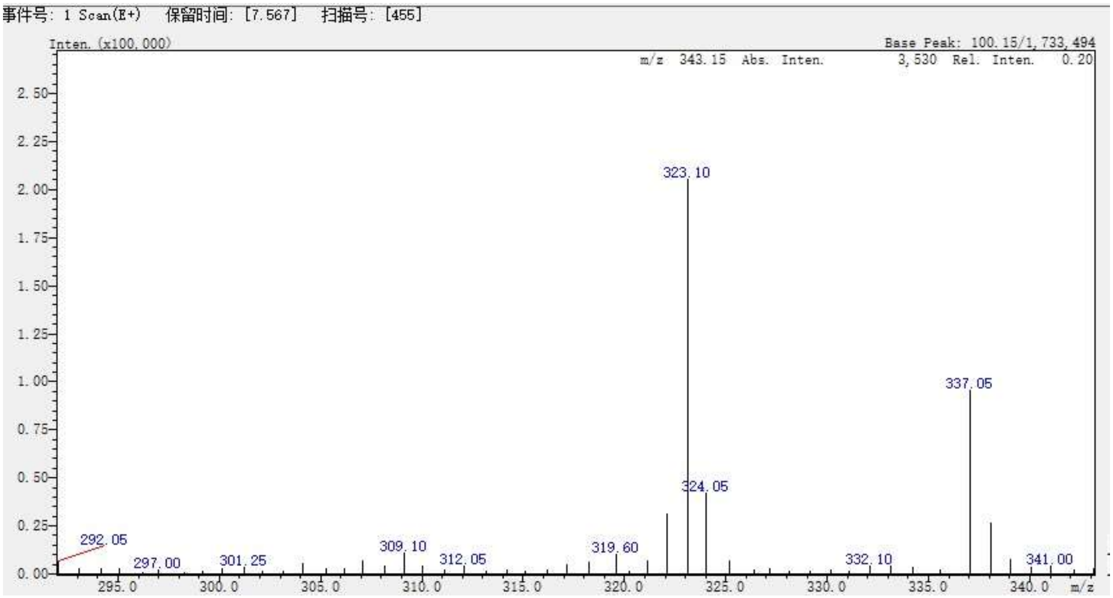

Figure S12. ESIMS spectrum of natural compound **4**.

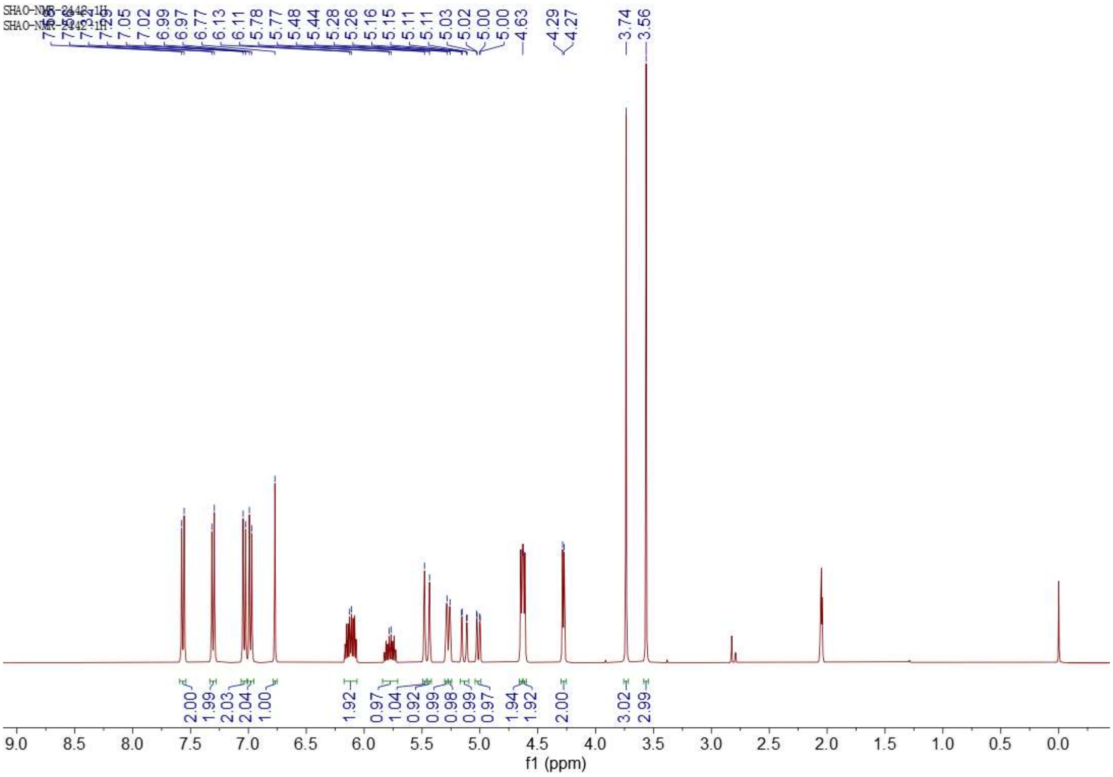

Figure S13. ^{1}H NMR (400 MHz, Acetone-d_6) spectrum of terphenyllin derivative compound **5.**

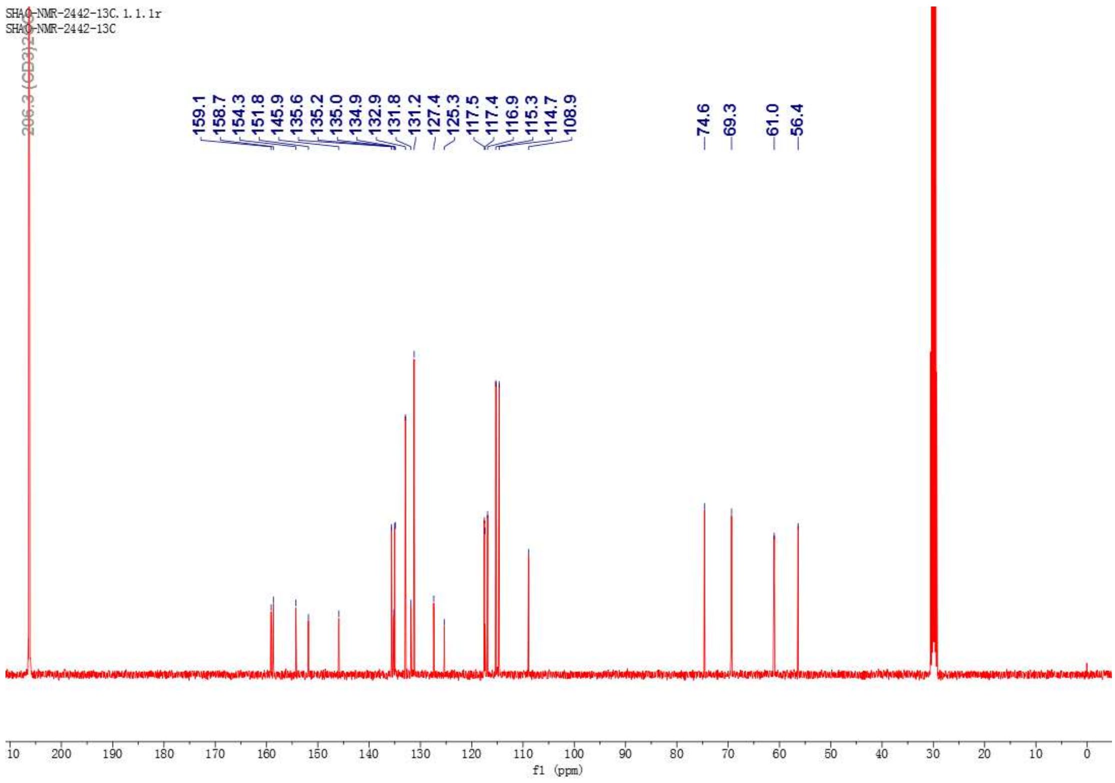

Figure S14. ^{13}C NMR (100 MHz, Acetone-d_6) spectrum of terphenyllin derivative compound **5.**

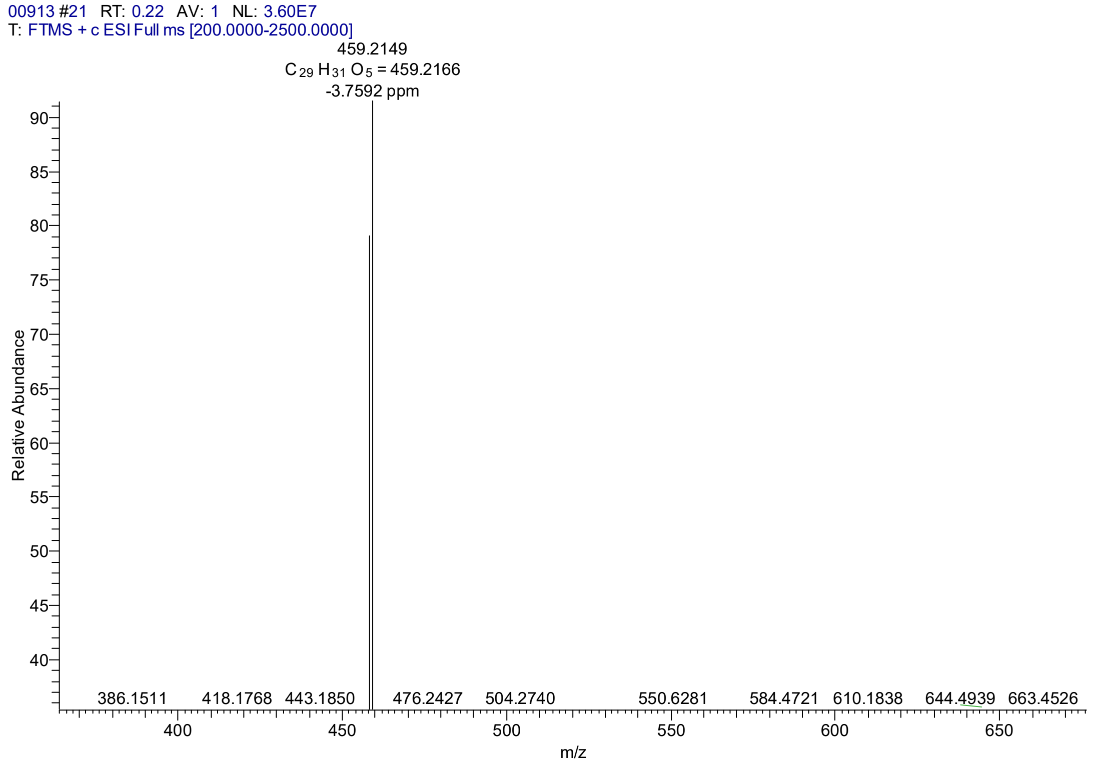

Figure S15. HRESIMS spectrum of terphenyllin derivative compound **5.**

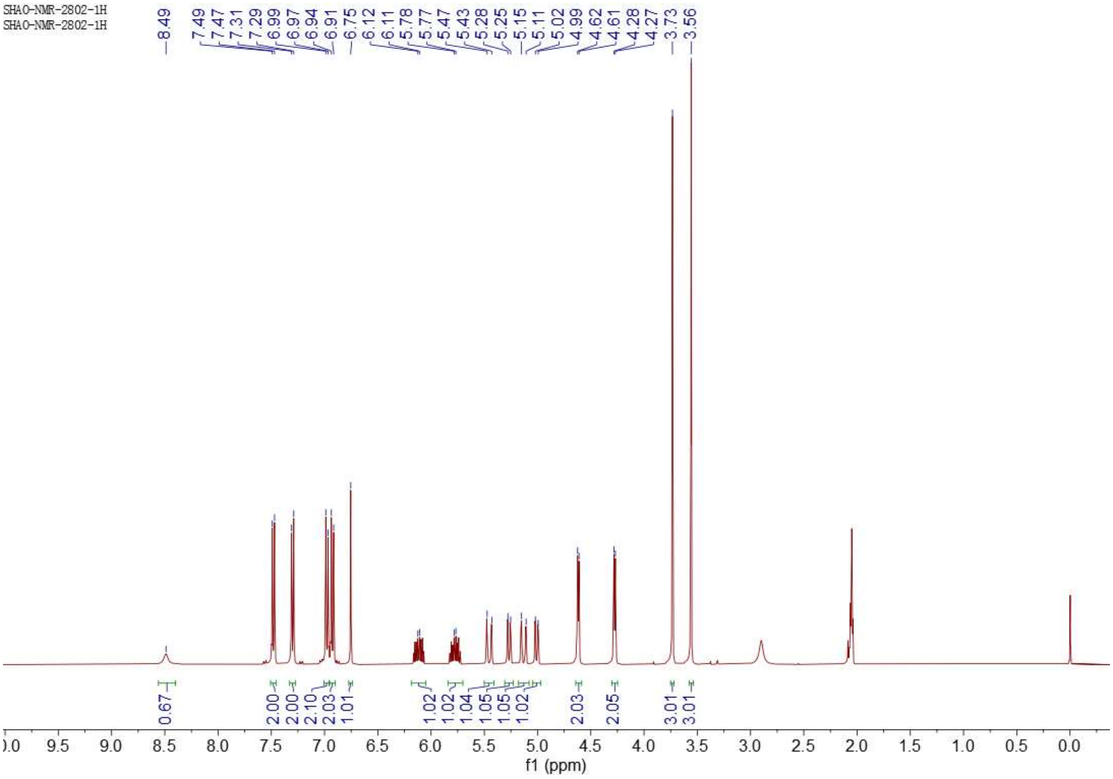

Figure S16. ^{1}H NMR (400 MHz, Acetone-d_6) spectrum of terphenyllin derivative compound **6**.

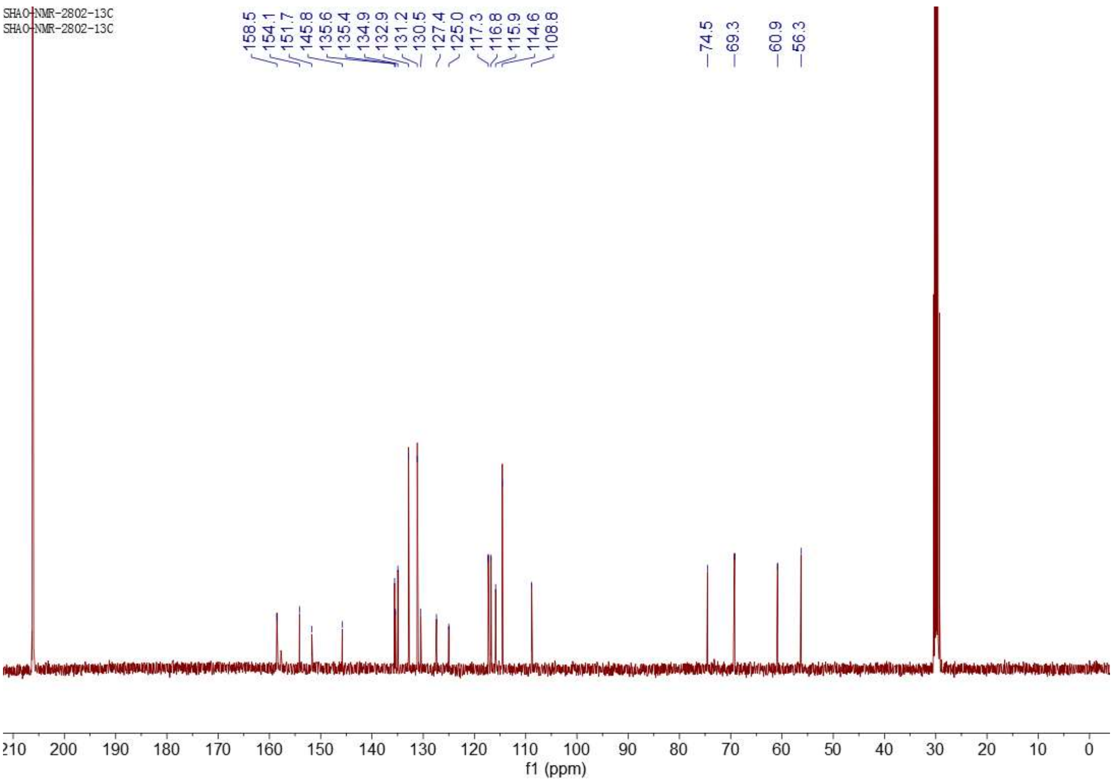

Figure S17. ^{13}C NMR (100 MHz, Acetone-d_6) spectrum of terphenyllin derivative compound **6**.

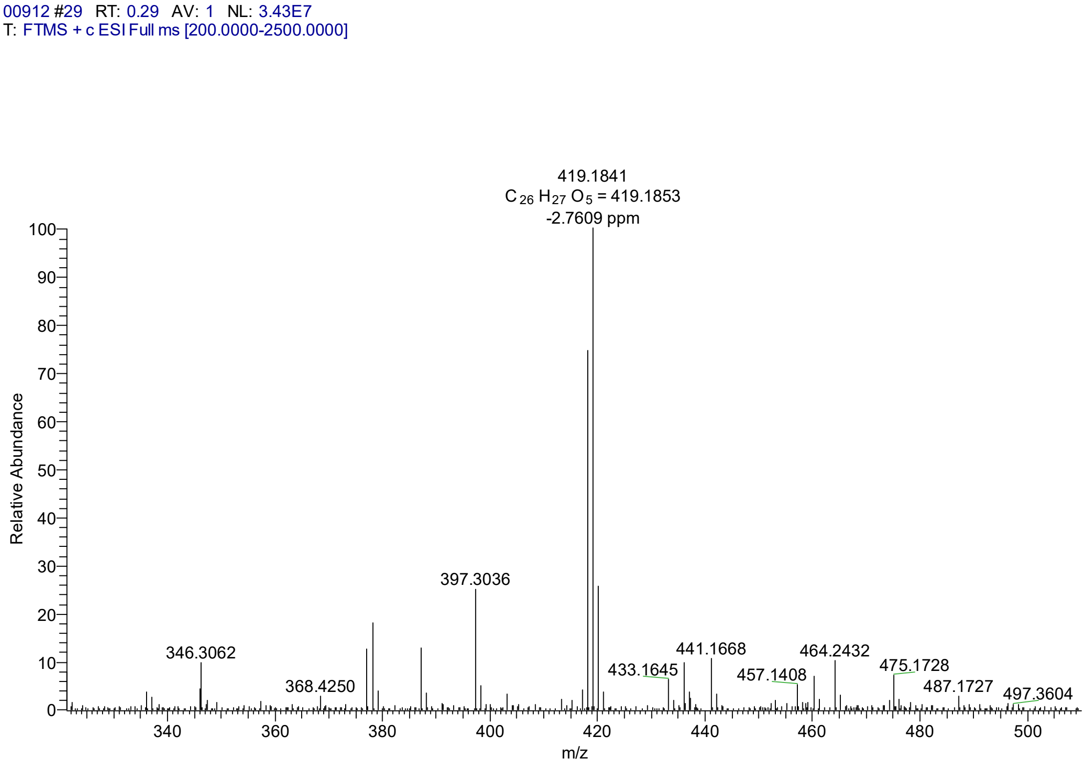

Figure S18. HRESIMS spectrum of terphenyllin derivative compound **6.**

4,4''-bis(allyloxy)-3',6'-dimethoxy-[1,1':4',1''-terphenyl]-2'-ol (**7**), White, amorphous powder; ^{1}H NMR (400 MHz, DMSO-d_6) δ 7.55 (2H, d, J = 8.8 Hz), 7.22 (2H, d, J = 8.8 Hz), 7.04 (2H, d, J = 8.8 Hz), 6.94 (2H, d, J = 8.8 Hz), 6.44 (1H, s), 6.08 (2H, s), 5.43 (2H, d, J = 14.2 Hz), 5.28 (2H,d, J = 10.5 Hz), 4.61 (4H, dd, J = 11.0, 5.2 Hz,), 3.65 (3H, s), 3.30 (3H, s). ^{13}C NMR (100 MHz, DMSO-d_6) δ 157.5 (C), 156.8 (C), 153.1 (C), 148.2 (C), 139.4 (C), 134.0 (C), 133.8 (CH), 132.3 (CH), 131.9 (CH×2), 130.5 (C), 129.8 (CH×2) , 126.4 (C), 117.5 (C), 117.4 (CH_2), 116.8 (CH_2), 114.5 (CH×2), 113.7 (CH×2), 103.0 (CH_2), 68.2 (CH_2), 60.2 (CH_3), 55.6 (CH_3). ESIMS m/z 419.10 [M + H]$^+$.

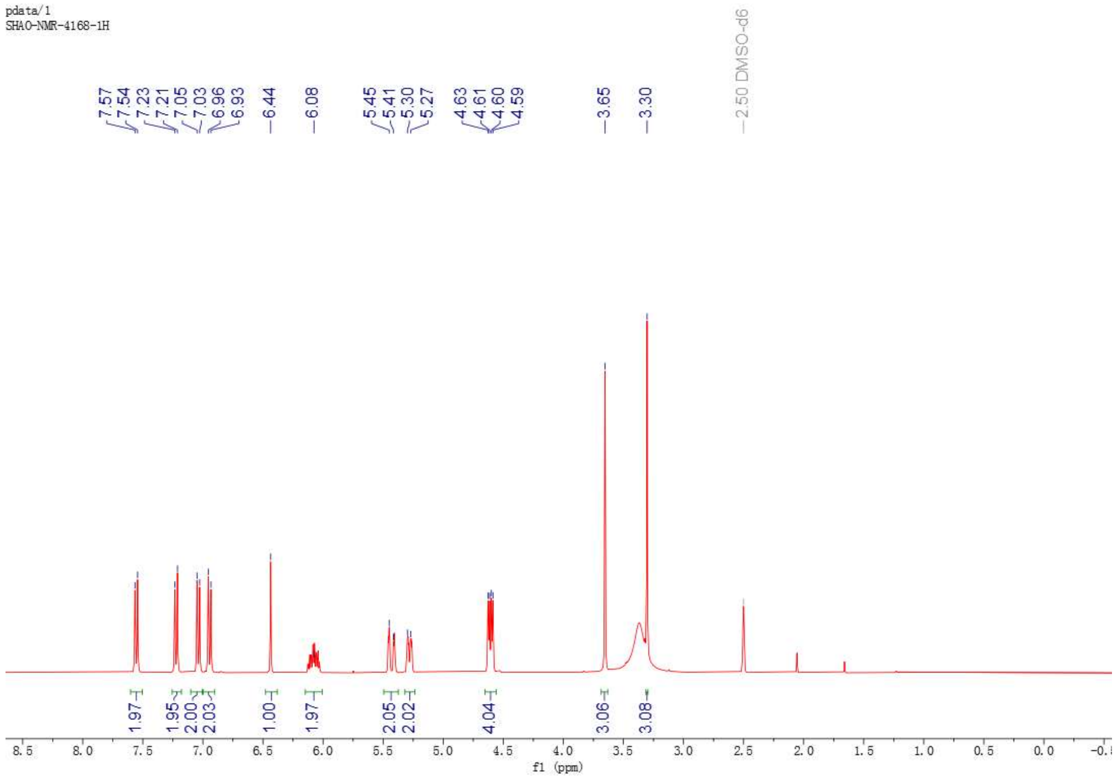

Figure S19. ^{1}H NMR (400 MHz,DMSO-d_6) spectrum of terphenyllin derivative compound **7**.

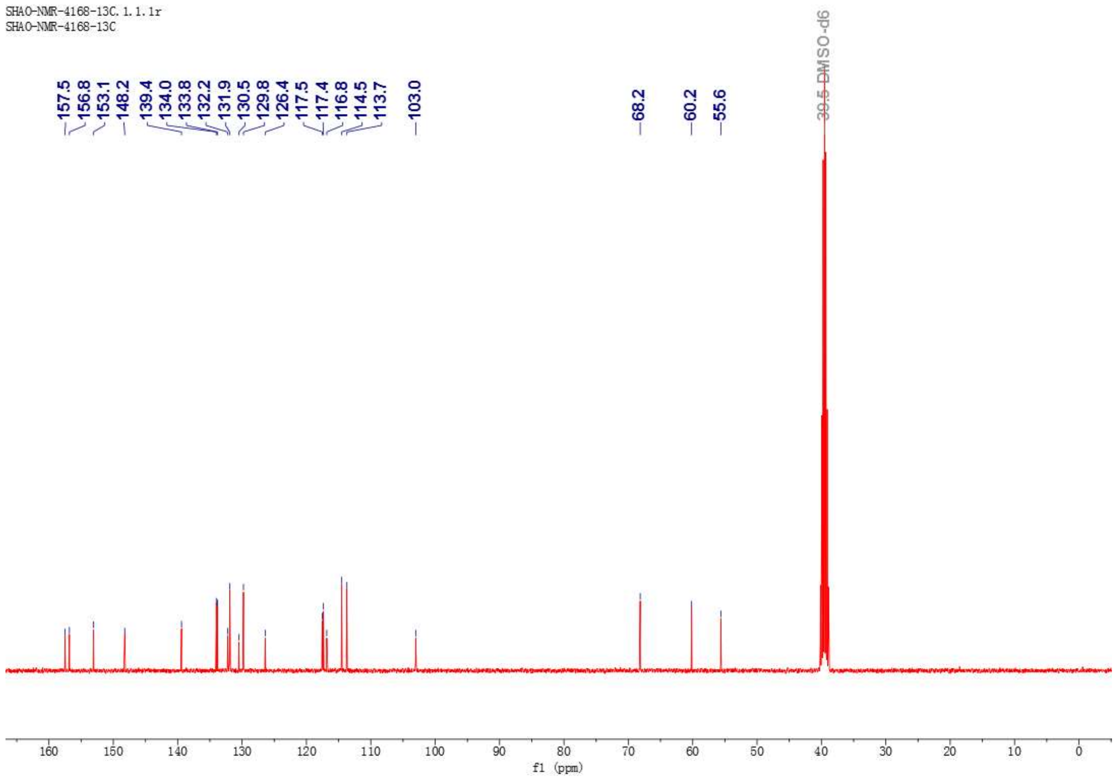

Figure S20. ^{13}C NMR (100 MHz, DMSO-d_6) spectrum of terphenyllin derivative compound **7**.

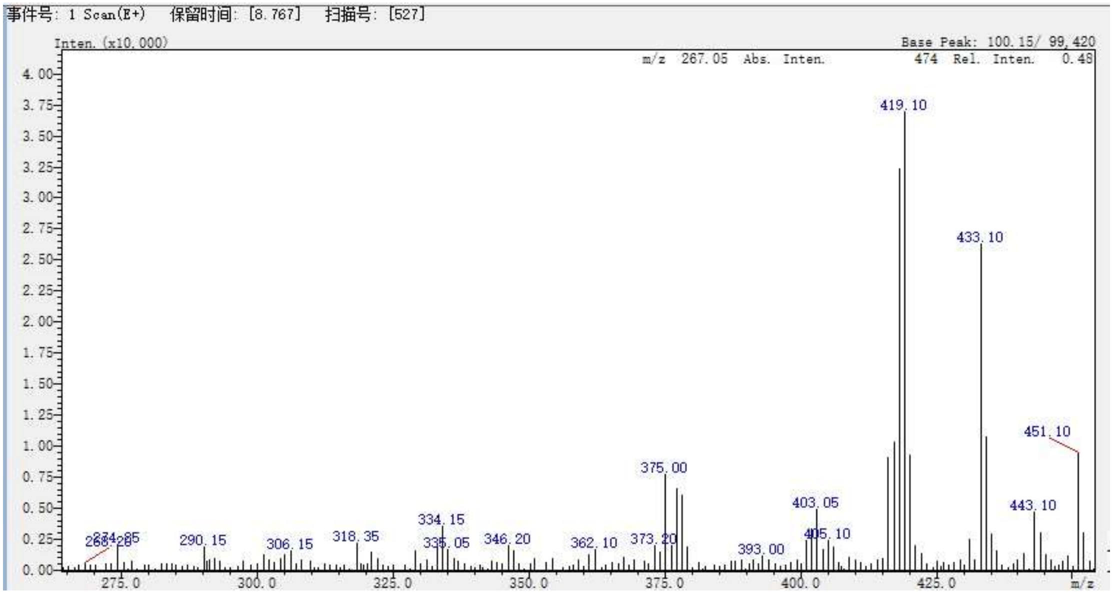

Figure S21. ESIMS spectrum of terphenyllin derivative compound **7**.

2′,4′′-bis(allyloxy)-3′,6′-dimethoxy-[1,1′:4′,1′′-terphenyl]-4-ol **8** (CHNQD-00824) was synthesized as Zhang described [17]. White, amorphous powder; ^{1}H NMR (400 MHz, $CDCl_3$) δ 7.53 (2H, d, *J* = 8.7 Hz), 7.31 (2H, d, *J* = 8.6 Hz), 7.00 (2H, d, *J* = 8.7 Hz), 6.88 (2H, d, *J* = 8.6 Hz), 6.68 (1H, s), 6.09 (1H, m), 5.76 (1H, m), 5.46 (1H, dd, *J* = 17.2, 1.6 Hz), 5.32 (1H, dd, *J* = 10.4, 1.6 Hz), 5.12 (1H, dd, *J* = 17.2, 1.6 Hz), 5.05 (1H, dd, *J* = 10.4, 1.6 Hz), 4.85 (1H, s), 4.61 (2H, d, *J* = 5.6 Hz,), 4.27 (2H, d, *J* = 5.6 Hz), 3.74 (3H, s), 3.62 (3H, s); ^{13}C NMR (100 MHz, $CDCl_3$) δ 158.1 (C), 154.6 (C), 153.2 (C), 150.9 (C), 145.1 (C), 134.4 (CH), 134.3 (CH), 133.4 (C), 132.3 (CH×2), 131.0 (C), 130.4 (CH×2), 126.3 (C), 124.4 (C), 117.9 (CH_2), 117.4 (CH_2), 114.9 (CH×2), 114.6 (CH×2), 108.1 (CH), 74.3 (CH_2), 69.0 (CH_2), 61.0 (CH_3), 56.3 (CH_3). ESIMS m/z 419.15 $[M + H]^+$.

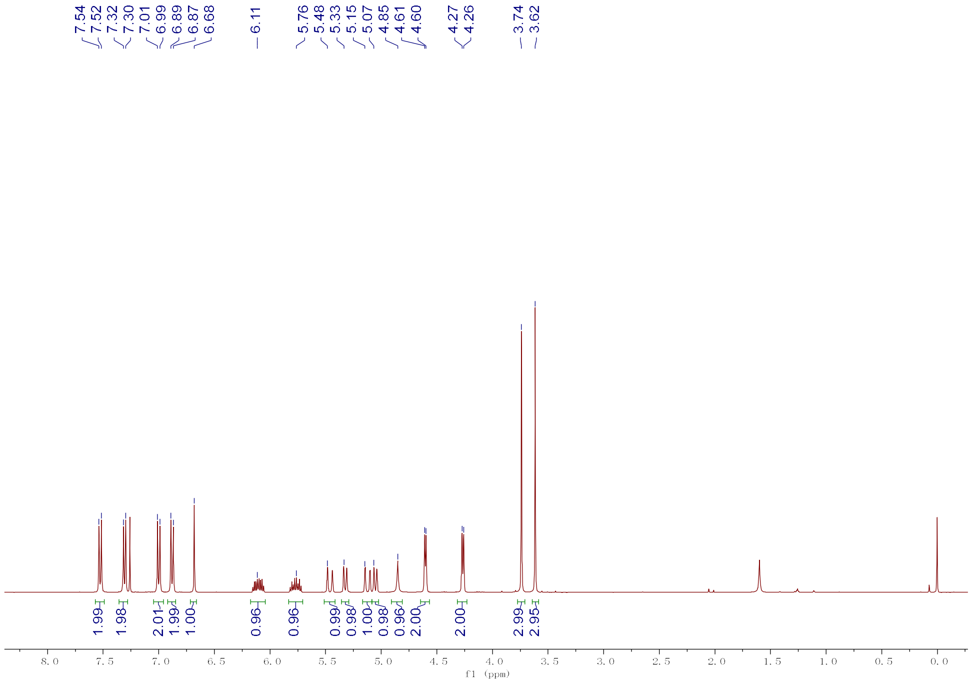

Fig. S22. ^{1}H NMR (400 MHz, $CDCl_3$) spectrum of terphenyllin derivative compound **8**.

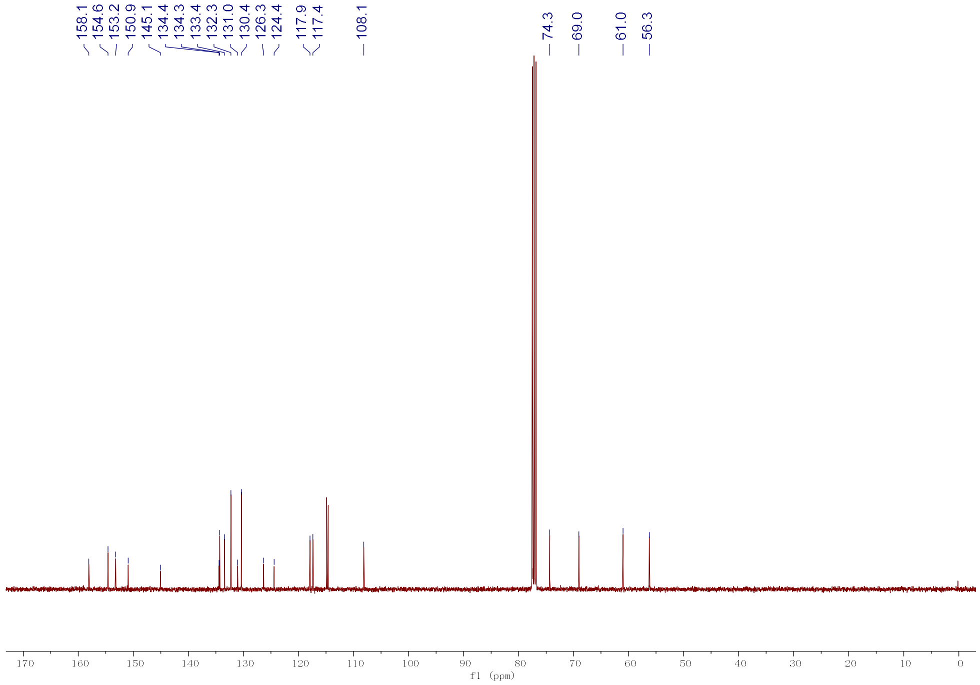

Fig. S23. ^{13}C NMR (100 MHz, $CDCl_3$) spectrum of terphenyllin derivative compound **8**.

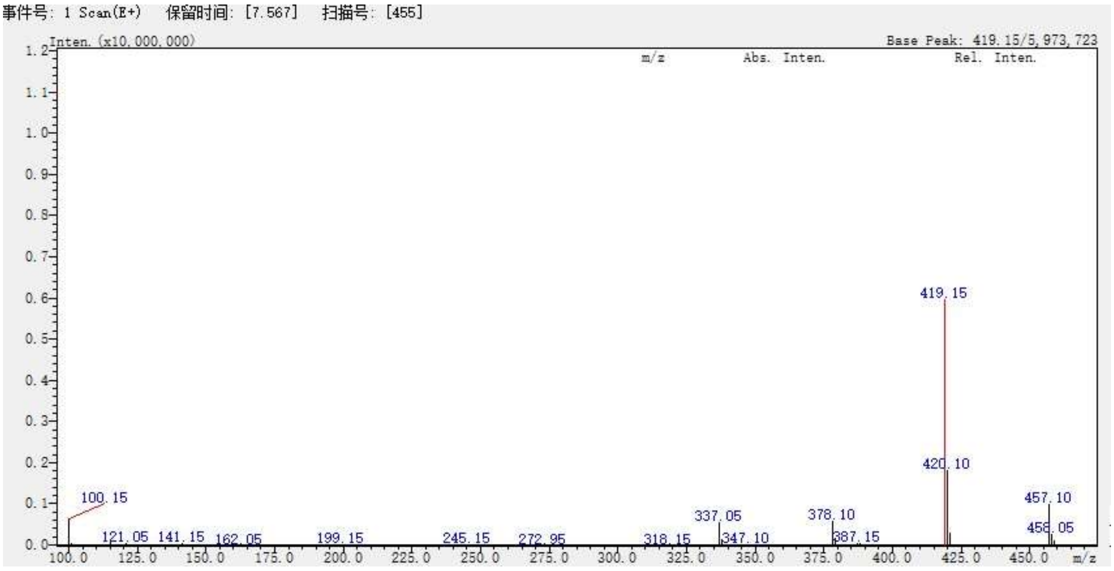

Figure S24. ESIMS spectrum of terphenyllin derivative compound **8**.

4-(allyloxy)-3',6'-dimethoxy-[1,1':4',1''-terphenyl]-2',4''-diol (**9**); White, amorphous powder; ^{1}H NMR (400 MHz, DMSO-d_6) δ 7.44 (2H, d, J = 8.7 Hz), 7.22 (2H, d, J = 8.7 Hz), 6.94 (2H, d, J = 8.7 Hz), 6.84 (2H, d, J = 8.7 Hz), 6.40 (1H, s), 6.07 (1H, s), 5.42 (1H, d, J = 19.0 Hz), 5.27 (1H, d, J = 8.9 Hz), 4.59 (2H, d, J = 5.3 Hz), 3.64 (3H, s), 3.30 (3H, s). ^{13}C NMR (100 MHz, DMSO-d_6) δ 156.9 (C), 153.0 (C), 148.2 (C), 139.3 (C), 134.0 (C×2), 132.6 (CH), 131.9 (CH×2), 129.7 (CH×2), 128.7 (C), 126.5 (C), 117.4 (CH_2), 116.5 (C), 115.2 (CH×2), 113.7 (CH×2), 102.9 (CH), 68.2 (CH_2), 60.1 (CH_3), 55.6 (CH_3). ESIMS m/z 379.10 $[M + H]^+$.

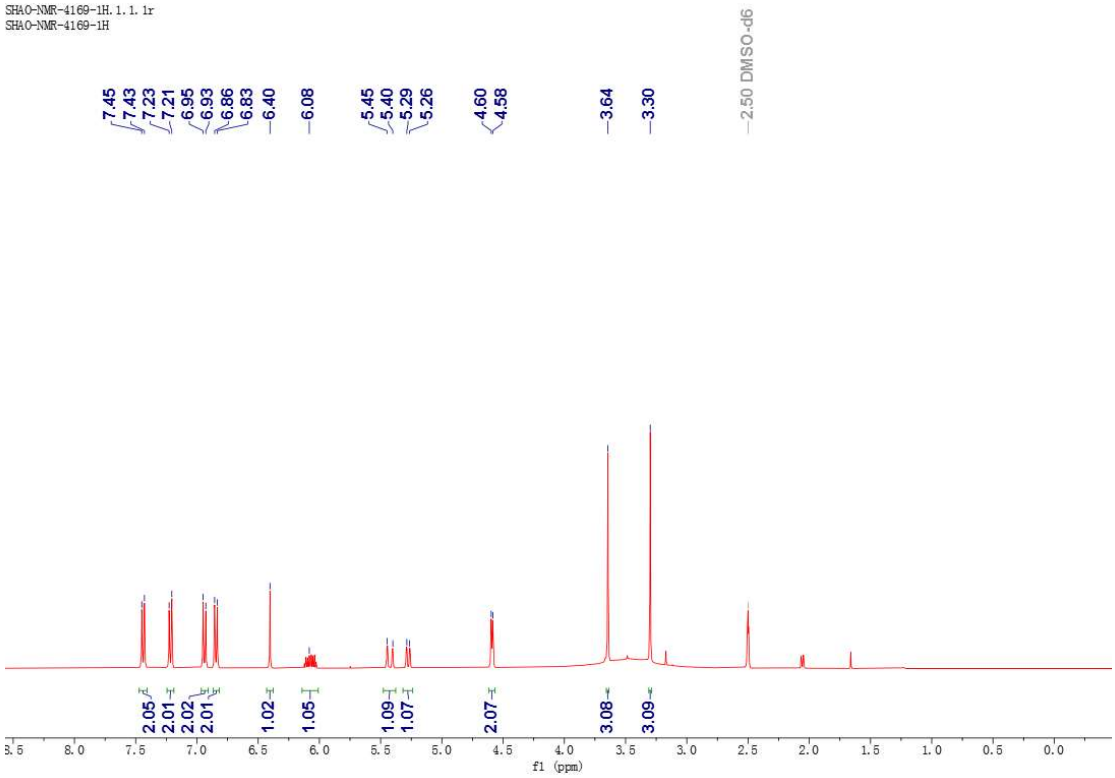

Figure S25. ^{1}H NMR (400 MHz,DMSO-d_6) spectrum of terphenyllin derivative compound **9**.

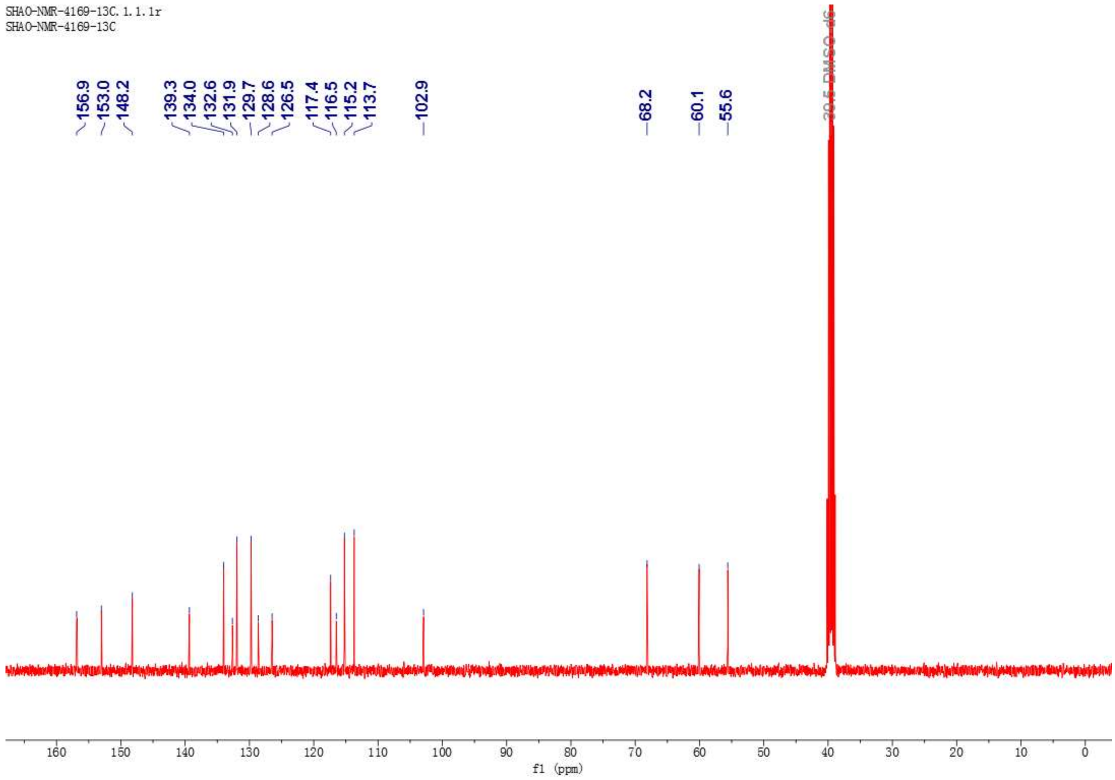

Figure S26. ^{13}C NMR (100 MHz, DMSO-d_6) spectrum of terphenyllin derivative compound **9**.

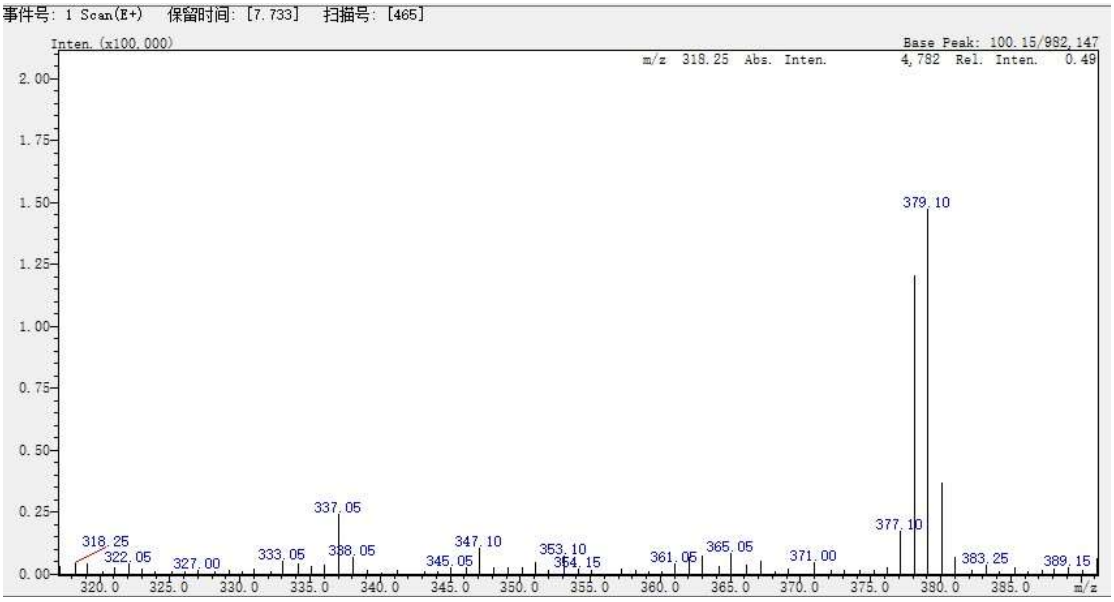

Figure S27. ESIMS spectrum of terphenyllin derivative compound **9**.

5-(allyloxy)-3',6'-dimethoxy-[1,1':4',1''-terphenyl]-2',4''-diol (**10**); White, amorphous powder; ^{1}H NMR (400 MHz, DMSO-d_6) δ 7.55 (2H, d, J = 8.8 Hz), 7.10 (2H, d, J = 8.6 Hz), 7.03 (2H, d, J = 8.8 Hz), 6.76 (2H, d, J = 8.6 Hz), 6.42 (1H, s), 6.08 (1H, s), 5.43 (1H, d, J = 19.0 Hz), 5.29 (1H, d, J = 10.6 Hz), 4.62 (2H, s), 3.64 (3H, s), 3.30 (3H, s). ^{13}C NMR (100MHz, DMSO-d_6) δ 157.5 (C), 156.0 (C), 153.2 (C), 148.2 (C), 139.4 (C), 133.8 (CH), 132.0 (C), 131.8 (CH×2), 130.6 (C), 129.8 (CH×2), 124.4 (C), 117.5 (CH_2), 117.3 (C), 114.5 (CH×2), 114.4 (CH×2), 103.0 (CH), 68.2 (CH_2), 60.2 (CH_3), 55.6 (CH_3). ESIMS m/z 379.10 $[M + H]^+$.

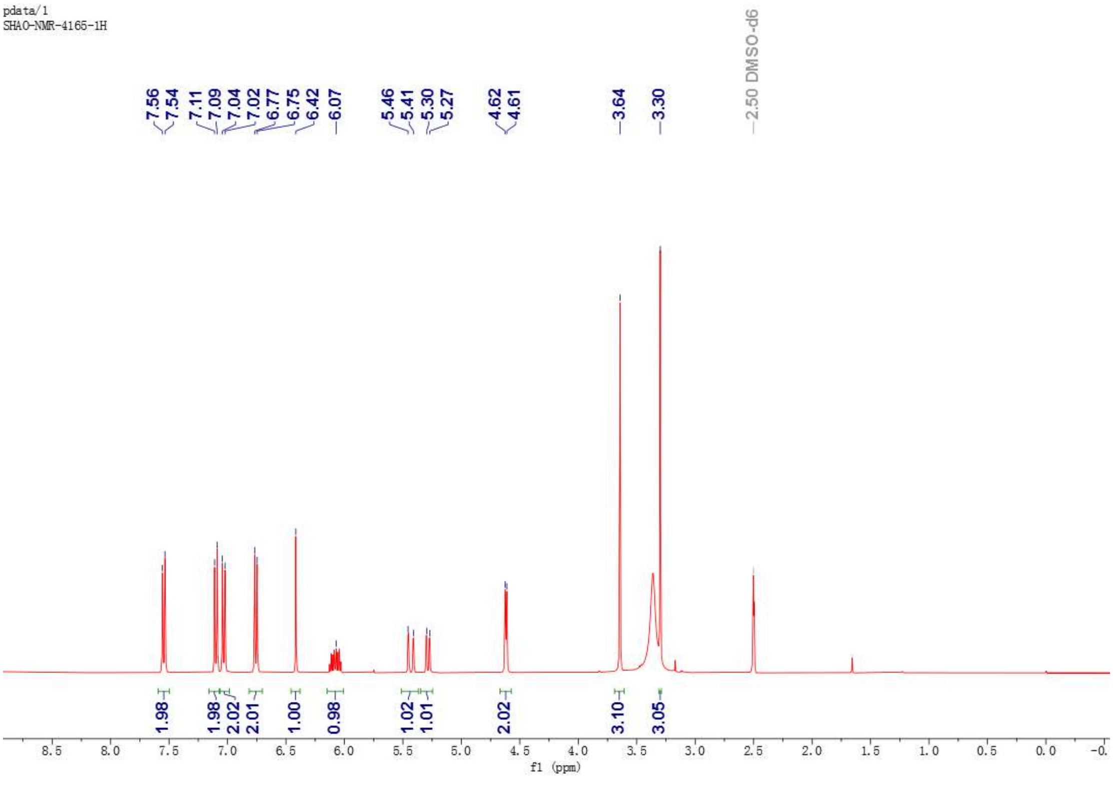

Figure S28. ^{1}H NMR (400 MHz,DMSO-d_6) spectrum of terphenyllin derivative compound **10**.

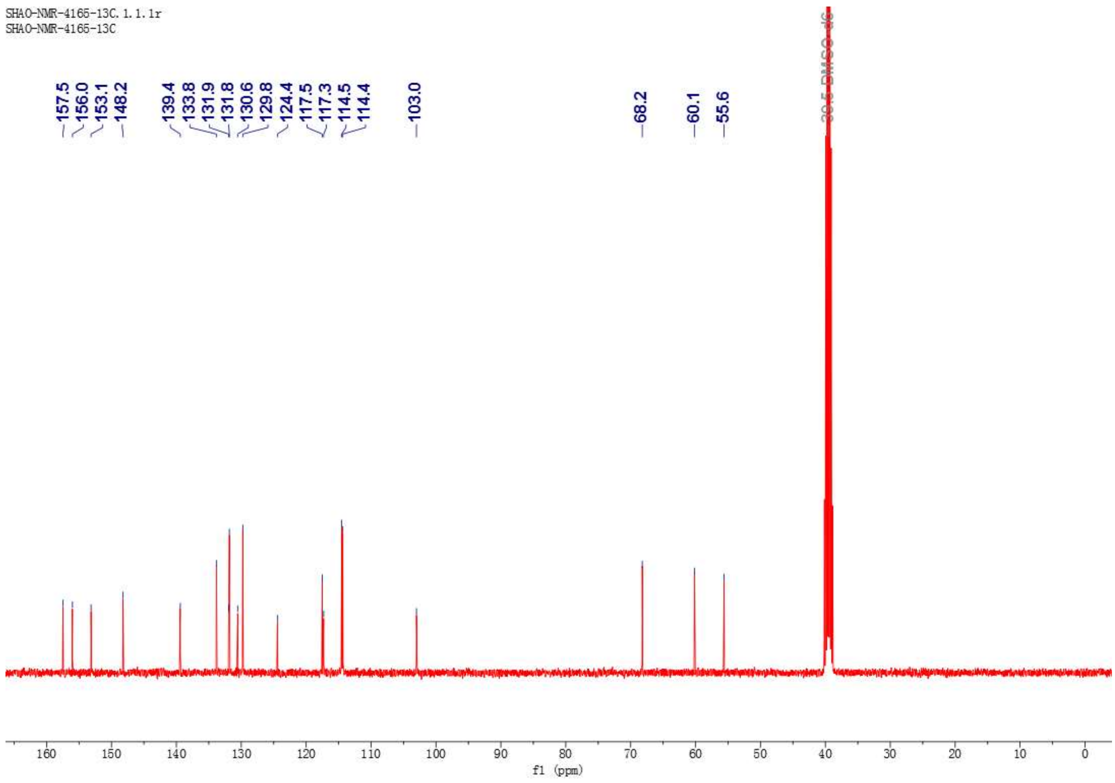

Figure S29. ^{13}C NMR (100 MHz, DMSO-d_6) spectrum of terphenyllin derivative compound **10**.

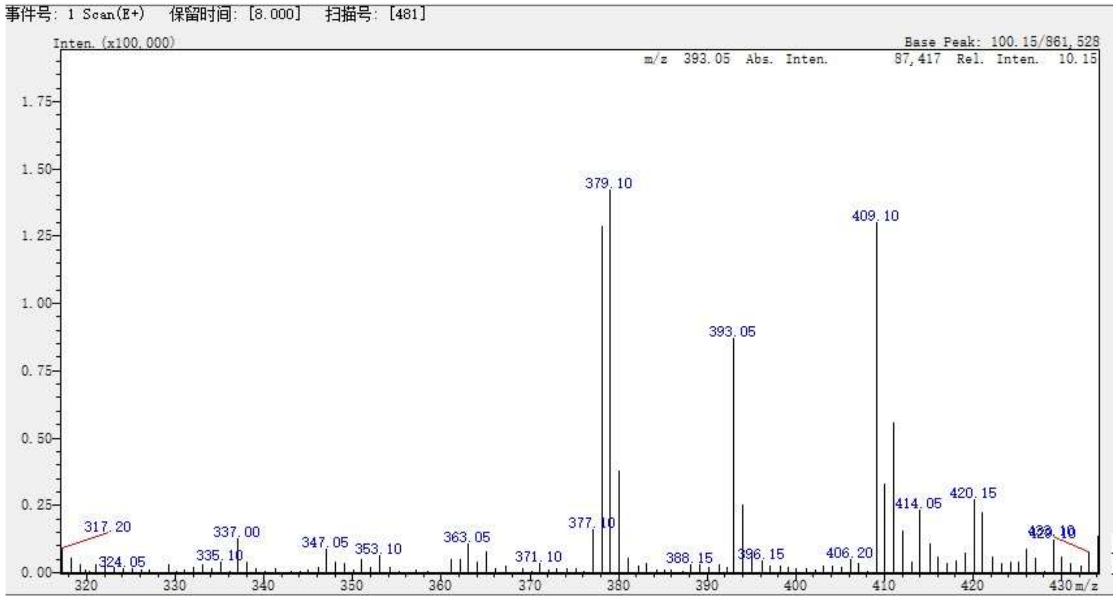

Figure S30. ESIMS spectrum of terphenyllin derivative compound **10**.

3'-(allyloxy)-2',5'-dimethoxy-[1,1':4',1''-terphenyl]-4,4''-diol (**11**); White, amorphous powder; ^{1}H NMR (400 MHz, DMSO-d_6) δ 9.45 (2H, s), 7.40(2H, d, J = 8.6 Hz), 7.10 (2H, d, J = 8.6 Hz), 6.84 (2H, d, J = 8.6 Hz), 6.78 (2H,d, J = 8.6 Hz), 6.68 (1H, s), 5.72 (1H, s), 5.17 – 4.98 (2H, m), 4.20 (2H, dt, J = 5.5, 1.5 Hz), 3.66 (3H, s), 3.49 (3H, s).^{13}C NMR (100 MHz, DMSO-d_6) δ 156.8 (C), 156.2 (C), 152. (C), 150.4 (C), 144.3 (C), 134.4 (C), 133.8 (CH), 131.6 (CH×2), 130.1 (CH×2), 128.6 (C), 124.1 (C), 123.9 (C), 116.7 (CH_2), 115.1 (CH×2), 114.5 (CH×2), 107.8 (CH), 73.4 (CH_2), 60.4 (CH_3), 55.8 (CH_3). ESIMS m/z 379.10 $[M + H]^+$.

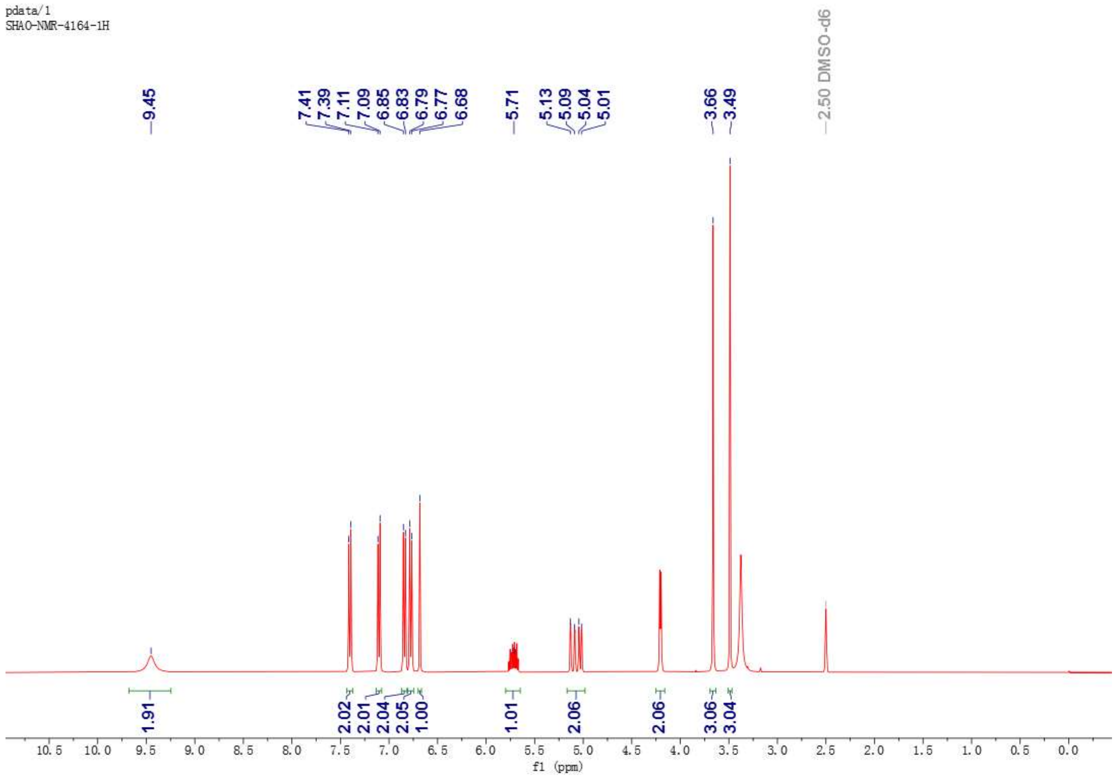

Figure S31. ^{1}H NMR (400 MHz,DMSO-d_6) spectrum of terphenyllin derivative compound **11**.

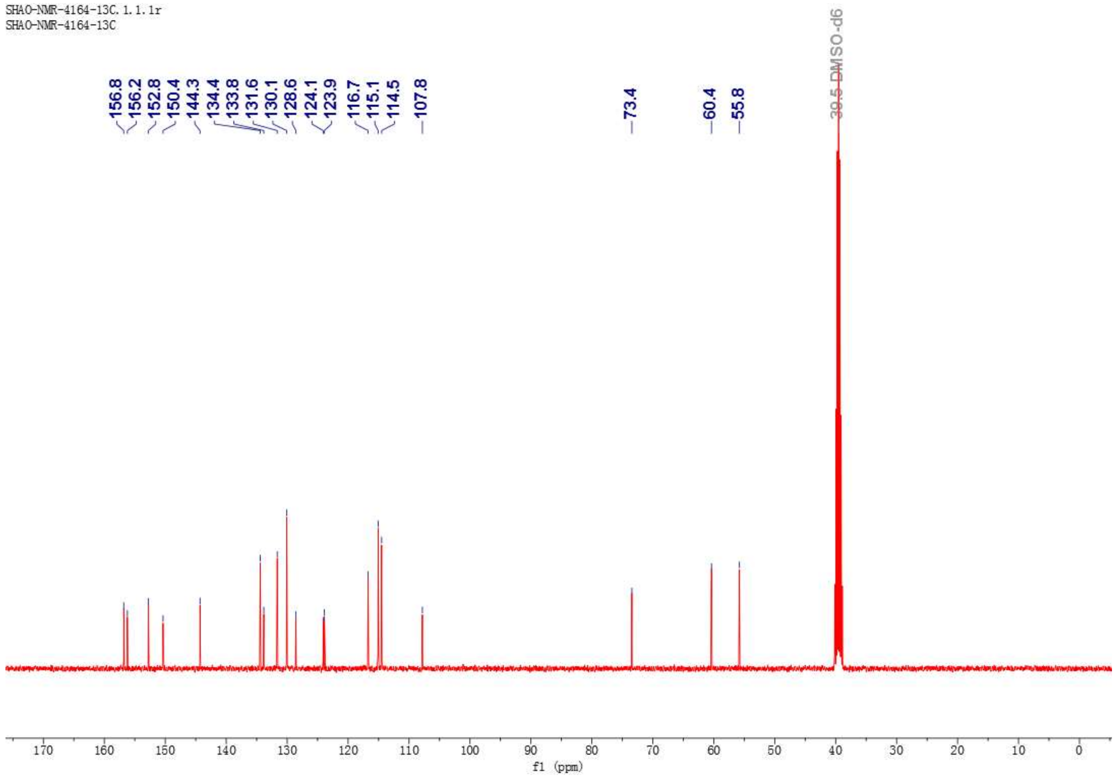

Figure S32. ^{13}C NMR (100 MHz, DMSO-d_6) spectrum of terphenyllin derivative compound **11**.

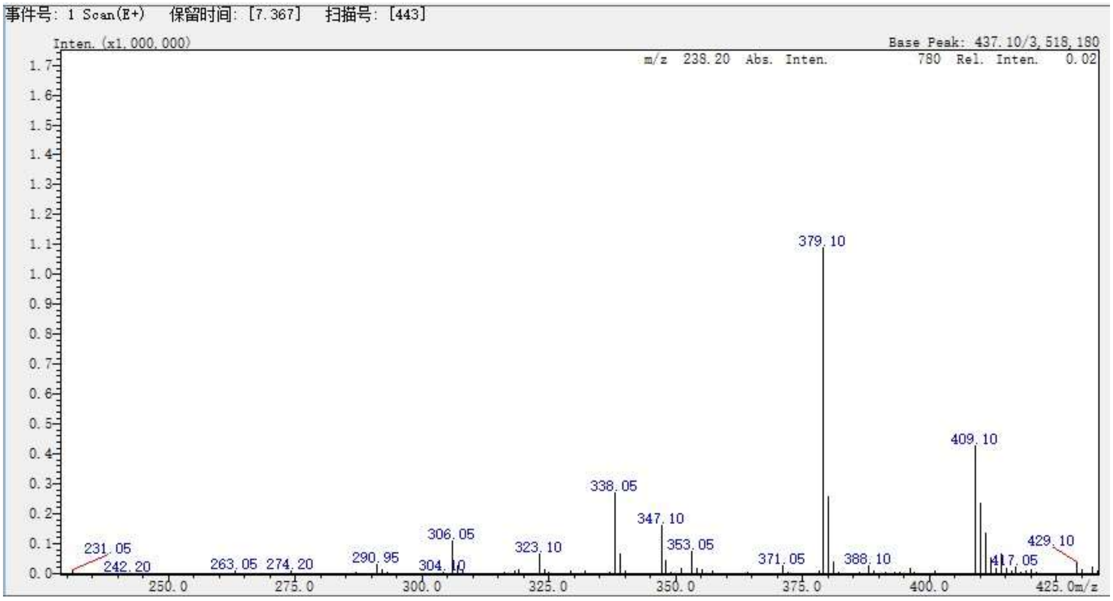

Figure S33. ESIMS spectrum of natural compound **11**.

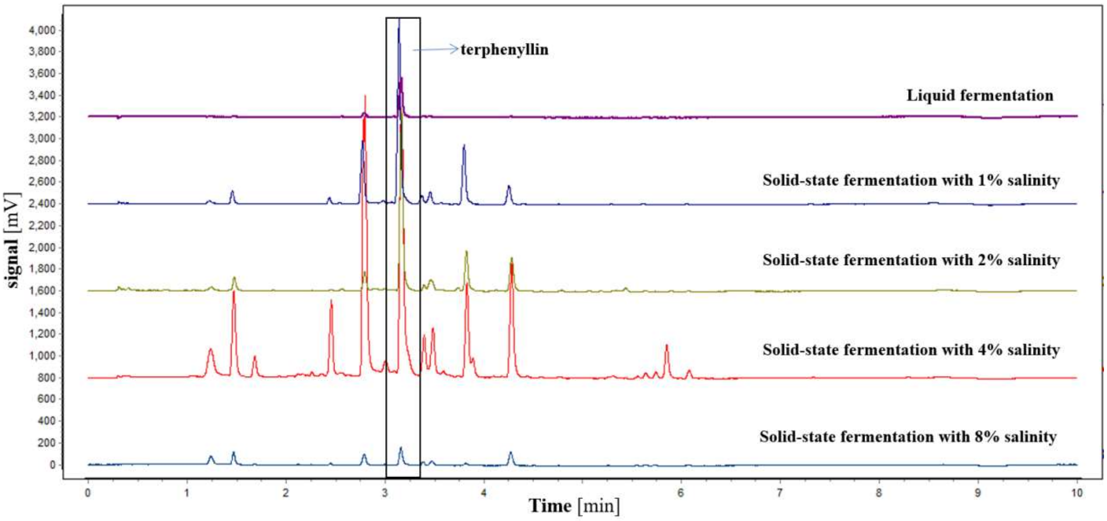

Figure S34. Fermentation yield comparison chart.

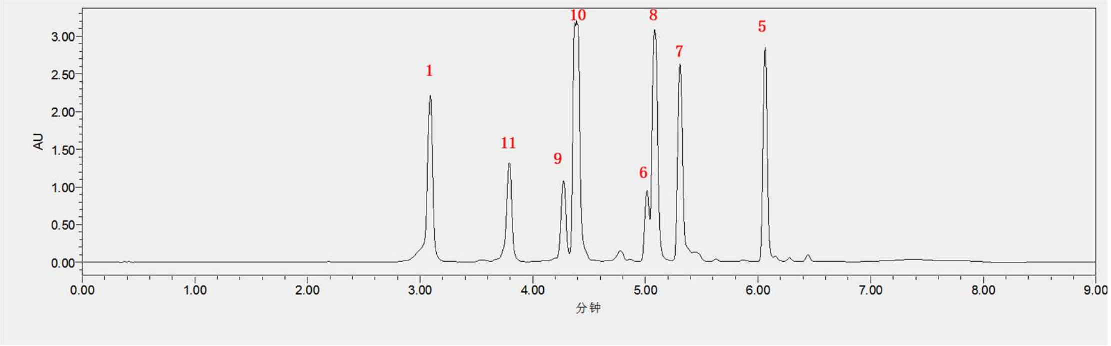

Figure S35. UPLC analysis of the reaction process of terphenyllin **1** treated with allyl bromide (2.5 equivalent). (The retention time of terphenyllin **1** and the seven derivatives **5-11** were 3.1 min

(terphenyllin 1), 3.8 min (derivative 11), 4.3 min (derivative 9), 4.4 min (derivative 10), 5.0 min (derivative 6), 5.1 min (derivative 8), 5.3 min (derivative 7), and 6.1 min (derivative 5), respectively.
